# Optimization of small coal pillar width and control measures in gob-side entry excavation of thick coal seams

**DOI:** 10.1038/s41598-024-74793-8

**Published:** 2024-10-07

**Authors:** Yan-ping Xue

**Affiliations:** 1 China Coal Technology and Engineering Group Shenyang Research Institute, Fushun, 113122 China; 2 State Key Laboratory of Coal Mine Safety Technology, Fushun, 113122 China

**Keywords:** Extra-thick coal seam, Roadway driving along next goaf, Deformation of surrounding rock, Destruction characteristics, Control strategy, Energy science and technology, Engineering

## Abstract

This study introduces an innovative optimized bolting support system specifically tailored for gob-side entry excavation in thick coal seams at a coal mine in southwestern Shandong, China. Employing theoretical analysis, numerical simulation, and field measurements, the research focuses on examining the failure characteristics of surrounding rock during gob-side entry excavation. The key innovation lies in the development of a 5-meter optimal coal pillar width, ensuring balanced stress distribution and structural integrity. Additionally, a lag time of at least 46 days between gob-side entry excavation and the upper working face retreat is recommended to mitigate roof subsidence and surrounding rock deformation. The optimized bolting support system, featuring increased bolt pretension, utilization of high-strength steel strips, and reinforcement of weak points, effectively reduces deformation of the roadway surrounding rock, meeting support requirements for normal production. This novel approach successfully addresses the support challenges in thick coal seam gob-side entry excavation, enhancing mining safety and resource recovery rates.

## Introduction

In high fully-mechanized longwall mining systems, the operational parameters—including large extraction heights and full-section longwall faces—intensify the mechanical stress on coal walls. This situation is exacerbated by the thick coal seams and extensive spans of roadways typical of such setups. These factors collectively contribute to significant overburden failure and pronounced rheological behavior of the surrounding rock mass, leading to marked mining-induced stresses. The resultant fractured coal walls present substantial challenges for support systems, complicating efforts to maintain structural integrity and operational safety. These challenges not only increase the technical complexity but also act as significant barriers to the widespread adoption of this advanced mining method^1–9^. To mitigate these effects, gob-side entry excavation is strategically implemented as a roadway layout method. This approach leverages the spatial configuration to naturally avoid the peak lateral abutment stress that typically accumulates on coal pillars. By doing so, it not only enhances the stability of the mining structure but also maximizes coal resource conservation. This strategic excavation method significantly improves recovery rates by allowing for more efficient coal extraction within the safety parameters of the mining environment. Such optimizations in the mining process provide crucial economic and environmental benefits, making gob-side entry excavation a valuable practice in modern mining operations^10–21^. Its advantages include reducing coal pillar width, enhancing coal extraction rates, and reducing abutment pressure within the internal stress field, which makes the surrounding rock easier to control^22–27^. To understand the deformation mechanism of the surrounding rock in gob-side entry excavation, it is essential to investigate the distribution pattern of lateral abutment stress in the coal body, establish a reasonable small coal pillar width, and define an appropriate lag time for the gob-side entry excavation. Analyzing the deformation characteristics of the surrounding rock yields field-based evidence crucial for optimizing the support layout for this configuration.

The success of gob-side entry excavation hinges on designing a reasonable coal pillar width. Selecting an appropriate width not only minimizes wastage of coal resources but also effectively avoids the peak abutment stress, ensuring successful implementation of gob-side entry excavation. The critical importance of the small coal pillar width in successful gob-side entry excavation manifests in two primary respects: (1) Stress Redistribution in Roadways: The small coal pillar width is crucial for redistributing stress within the roadway’s surrounding rock. (2) Roadway Integrity Maintenance: The dimension of the small coal pillar is essential for preserving the overall stability and integrity of the surrounding rock^28–34^. Researchers globally have achieved significant progress in determining the dimensions of small coal pillars and managing large-section roadways, offering valuable insights into these challenges.

Yang Jiping et al^35^., applied the theory of large and small structures in surrounding rock to theoretically determine the optimal size of small coal pillars for gob-side entry excavation. Zhang Kexue et al^36^., based on geological conditions and using the limit equilibrium theory, studied the lateral stress distribution pattern in the goaf of the upper segment. They verified the method through numerical simulation and field experiments to determine the appropriate small coal pillar size. Xie Guangxiang et al^37^. used FLAC3D to simulate the surrounding rock deformation of the caving mining roadway at different coal pillar widths and found that the failure patterns differed between the solid coal side and the coal pillar side. Feng Jicheng et al^38^. developed a formula to calculate the fracture location on the goaf side of the upper working face using the stress limit equilibrium theory. Using numerical simulation, they elucidated the stress distribution characteristics at the edge of the coal seam. Zheng Xigui et al^39^. analyzed the stress distribution and stability of small coal pillars during roadway development and mining, studying how different pillar sizes impact stress evolution. Sun Dongfei et al^40^. proposed an integrated surrounding rock control technology to address issues with gob-side entry excavation, such as rock fractures, mining disturbances, narrow coal pillars, and the roadway support system. Sun Fuyu et al^41^., using a combination of field research, theoretical analysis, and numerical simulation, investigated the deformation and failure characteristics of the surrounding rock in narrow coal pillar gob-side entries, along with corresponding control techniques. Zhang Guangchao et al^42^. conducted a FLAC3D numerical analysis in the 20,103 section of the Wangjialing Coal Mine and concluded that a reasonable coal pillar width ranges from 6 to 10 m.

This study, through a comprehensive analysis of a fully mechanized gob-side entry excavation in a thick coal seam, endeavors to ascertain the optimal width for the small coal pillar and the appropriate lagging excavation time. It meticulously examines the challenges and control principles pertaining to supporting the surrounding rock during gob-side entry excavation in the context of high-thickness, fully mechanized mining. With the aim of enhancing the stability and safety of the excavation process, an optimized roadway support plan is proposed, emphasizing the strategies of “increasing bolt pretension, utilizing high-strength steel strips, and reinforcing vulnerable points.” This study ultimately seeks to ensure robust control over the gob-side roadway, thereby mitigating scheduling constraints and fostering efficient and safe mining operations.

### Engineering geological conditions

**Fig. 1:**
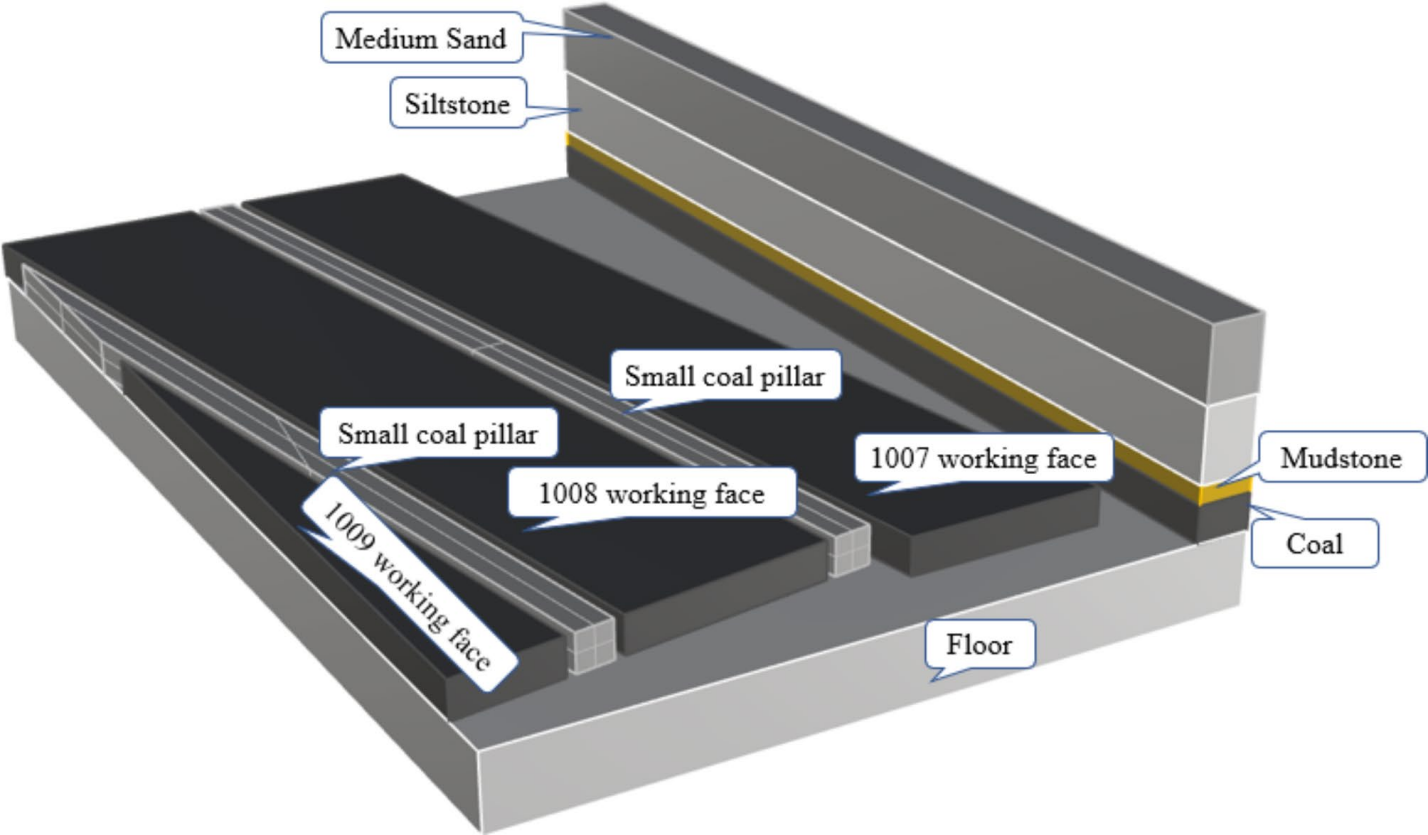
Relative position of the working face.

The 1007 working face of a certain mine is the first fully mechanized longwall face after the mine’s upgrade. Its strike length is 724 m, and its dip length is 175 m. The coal seam has an average thickness of 5.5 m and a dip angle ranging from 5 to 9 degrees. The elevation range of the coal seam floor is -455.3 to -540.0 m. The 1008 and 1001 working faces are located to the north and south of the 1007 face, respectively. The 1001 working face has been mined out, while the 1008 working face is a trial working face. Figure 1 describes the distribution of the top and bottom rock layers of the coal seam and the relative positional relationship between the working faces.

**Fig. 2:**
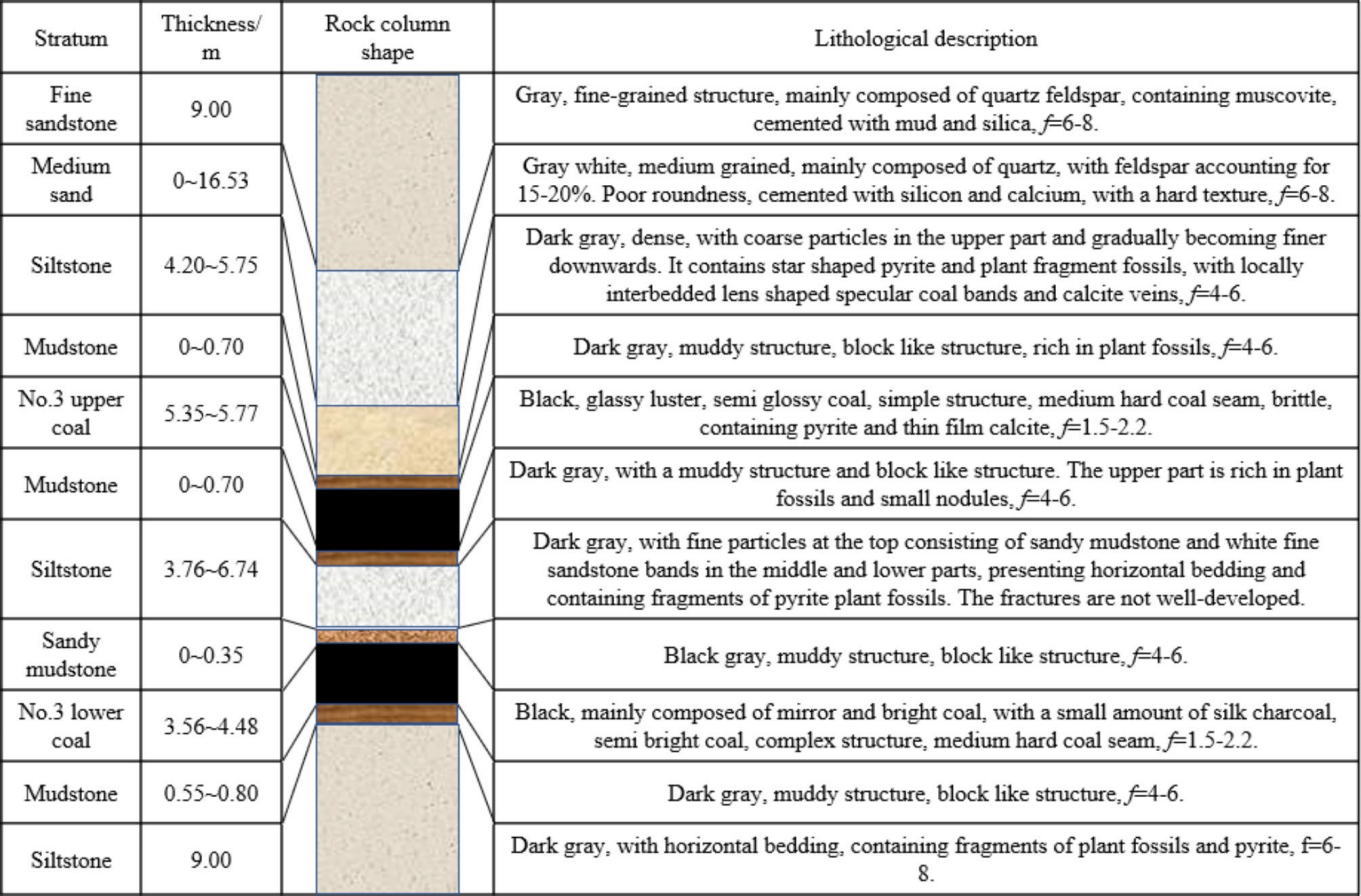
Histogram of the top and bottom plates of the coal seam.

The 3 Upper Coal Seam exhibits a thickness ranging from 5.35 to 5.77 m, with an average of 5.50 m. The coal body contains well-developed fissures, which exhibit a banded structure. Based on the conditions revealed from adjacent mined working faces and excavation roadways, large-scale geological structures have not affected the coal seam’s structure. Figure 2 presents the distribution of the top and bottom rock strata of the coal seam, the thickness of the rock strata and the lithologic description of each rock strata.

## Study on the optimal position and timing for gob-side entry excavation

### Study on the optimal position for gob-side entry excavation

Identifying the low-stress region in the lateral coal body and determining the optimal coal pillar width require monitoring the stress within the coal under specific conditions. The direct measurement of lateral abutment pressure distribution behind the goaf poses challenges due to mining constraints. Nevertheless, given the similarities between lateral and advancing abutment pressures, the lateral abutment pressure distribution can be inferred by monitoring the advancing abutment pressure distribution.

#### Monitoring plan

**Fig. 3:**
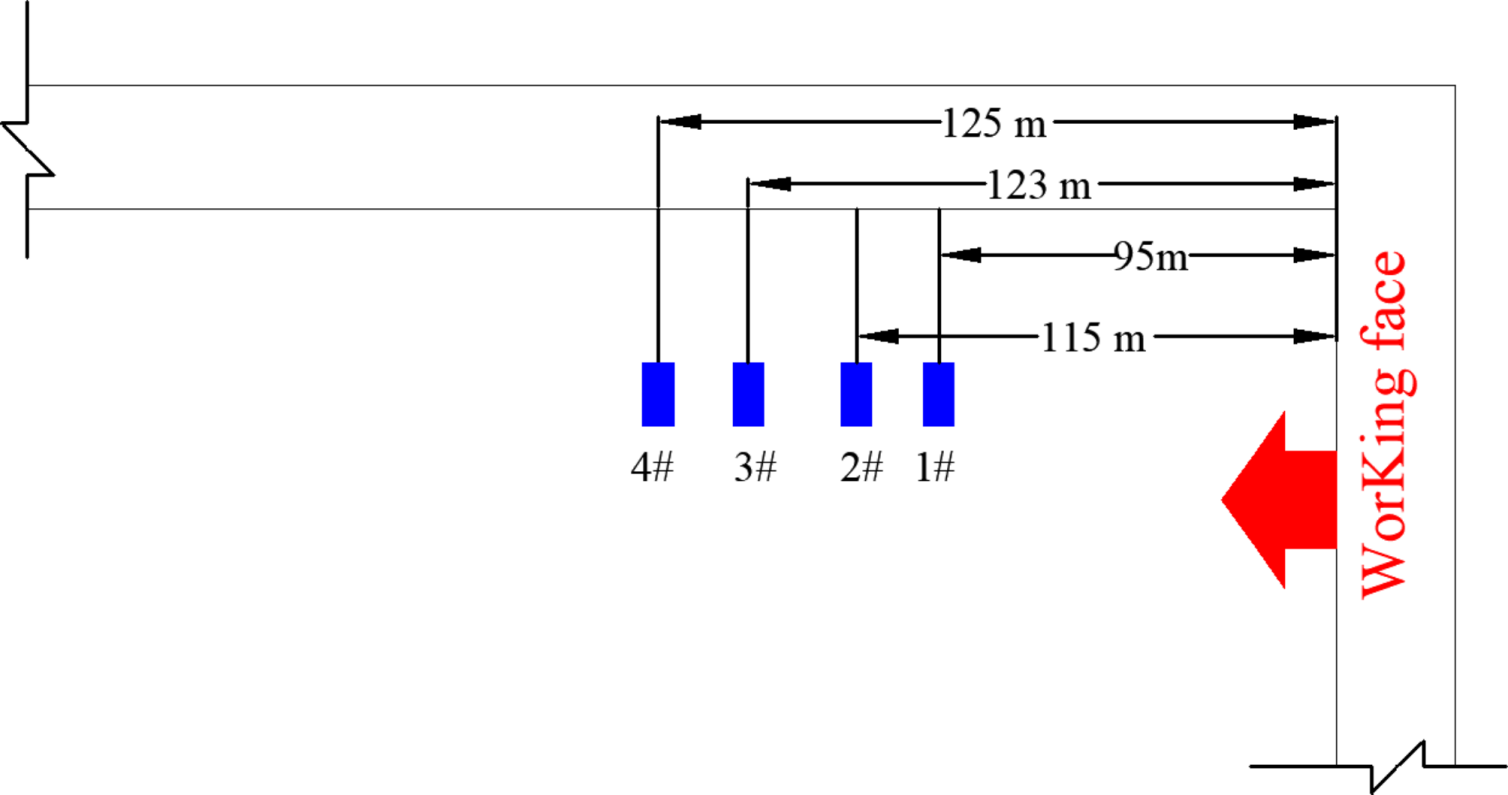
A schematic diagram of the layout of a borehole stress meter.

Four observation points were established approximately 100 m from the working face coal wall, with each borehole reaching a depth of 6 m. For the detailed arrangement, see Fig. 3.

#### In-situ analysis of borehole stress meter measurements

**Fig. 4:**
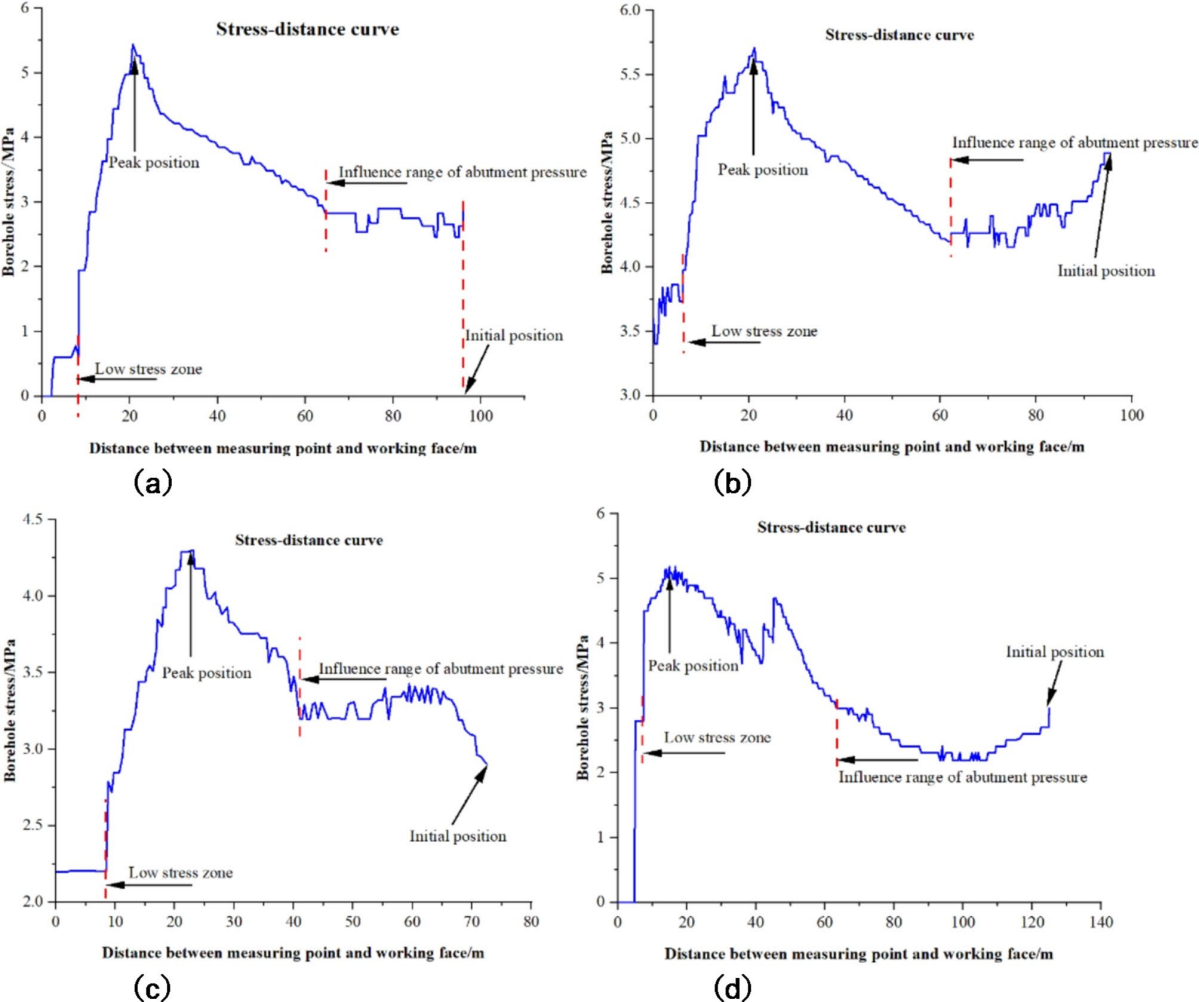
Results of borehole stress observation. (**a**) Observation results of borehole #1 stress meter. (**b**) Observation results of borehole #2 stress meter. (**c**) Observation results of borehole #3 stress meter. (**d**) Observation results of borehole #1 stress meter.

The stress curves recorded by the borehole stress meters relative to the working face position are depicted in Fig. 4 (a) to (d). The stress meter in Borehole #3 was installed 123 m away from the working face, but a segment of data is missing from the earlier stages. Actual observations began at a point 73 m from the working face.

The initial pressure recorded in borehole #1 was 3.0 MPa. As the working face advances, the stress gradually increases, reaching a peak of 5.5 MPa. Following the peak, the stress begins to diminish. Prior to the working face reaching a distance of 8.4 m, the stress decline curve is relatively gentle. Beyond this point, the stress value decreases in a stepwise fashion, ultimately stabilizing at 2.4 MPa. The initial pressure recorded in borehole #2 was 5.0 MPa. With the retreat of the working face and compaction of the coal body, the stress initially escalates sharply, followed by a more gradual increase. The stress reaches a maximum value of 5.7 MPa at 21.5 m from the working face, then starts to decrease. Up to 9.3 m from the working face, the decline curve fluctuates significantly but with a small amplitude. Beyond this point, the stress decreases in a stepwise fashion, settling at 3.8 MPa. The initial pressure recorded in borehole #3 was 2.9 MPa. The stress reaches a peak of 4.28 MPa at 22.7 m from the working face coal wall. After reaching the peak, the stress starts to decrease. Before reaching 8.7 m from the working face, the decline curve is relatively gentle. Beyond this point, the stress value drops in a step-like manner, eventually stabilizing at 2.2 MPa. The initial pressure recorded in borehole #4 was measured 2.7 MPa. At a distance of 43.5 m from the working face coal wall, stress values began to decrease, potentially due to coal-rock crushing within the borehole, inducing fluctuations. Following compaction, the stress subsequently rose, peaking at 15 m from the working face coal wall. Subsequently, the stress began to decline. The decline was gentle until 7.5 m from the working face, after which it sharply dropped to 2.8 MPa in a stepwise fashion. Eventually, the stress plummeted to zero when the stress meter was positioned 2.8 m from the working face.

**Table 1:** Influence range of support pressure determined according to stress of borehole.

Stress meter number	1#	2#	3#	4#	Average
Significant Influence Range of Abutment Pressure /m	63.80	61.50	40.8	63.20	57.30
Distance from the Working Face to the Peak Abutment Pressure /m	22.50	21.50	22.7	15.00	20.43
Location of the Low-Stress Zone from the Working Face /m	8.40	9.30	8.70	7.50	8.50

Table 1 shows the monitoring results obtained at each station and their average values. Comprehensive analysis of the monitoring results from the four borehole stress meters reveals that the significant influence range of advanced abutment pressure is within 57.3 m in front of the working face coal wall, with the peak pressure located 23.6 m ahead of the working face coal wall. The low-stress zone is identified as being approximately 8.5 m from the working face coal wall. Consequently, it can be reasonably inferred that the lateral coal body at the working face also exhibits a low-stress zone extending roughly 8.5 m.

#### Numerical simulation study of reasonable size of small coal pillar

**Fig. 5:**
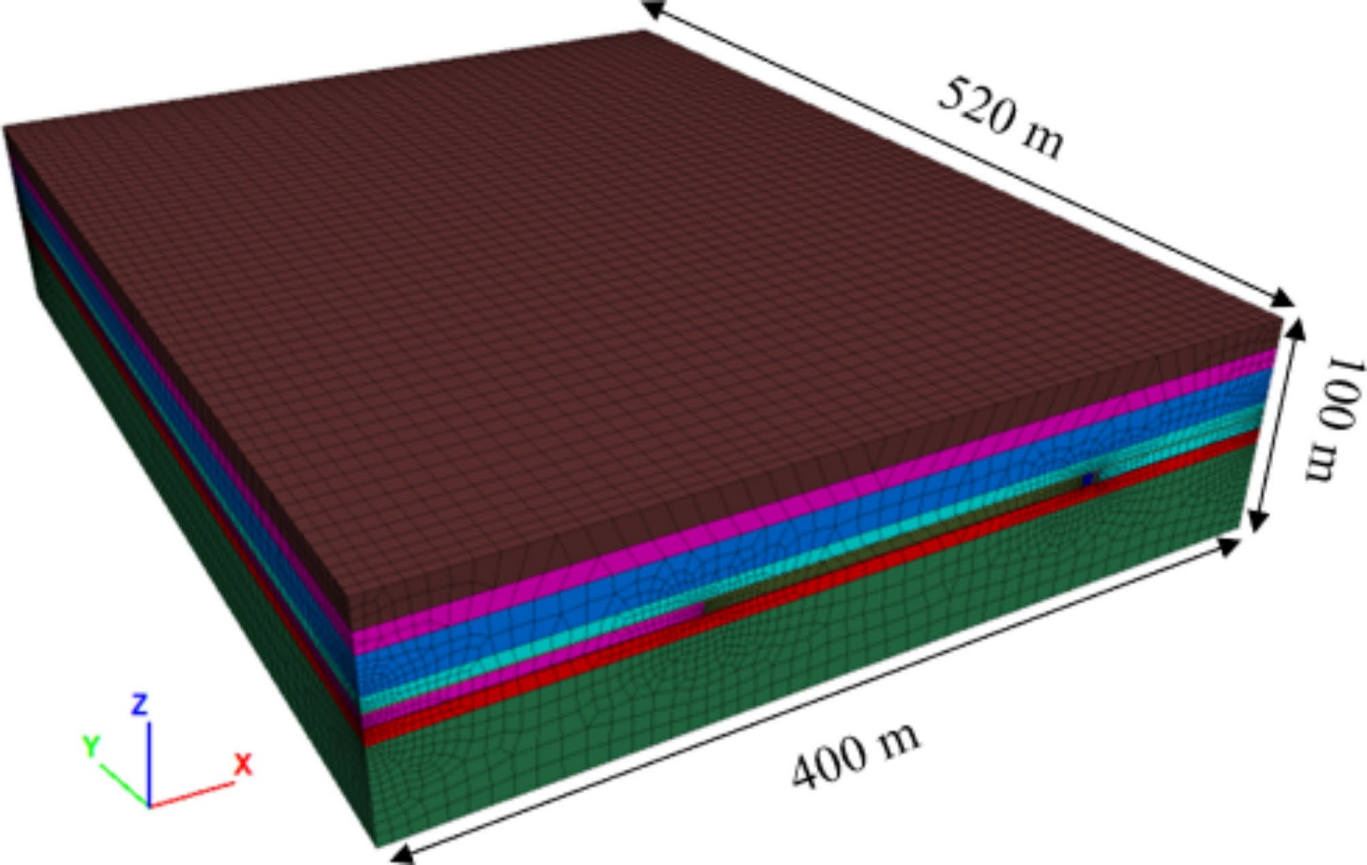
Numerical model.

To determine the appropriate size of small coal pillars for gob-side entry excavation, a numerical model (Fig. 5) was established based on the actual conditions of the mine. The model considered both computational accuracy and speed, with the dip direction set as the X-axis (400 m long), the strike direction as the Y-axis (520 m long), and the vertical direction as the Z-axis (100 m high). The overlying strata, coal seam, and floor lithology were simplified and divided according to engineering geological types (formation groups). Each stratum in the model was classified based on the stratigraphic column, and layers with similar mechanical properties and small thicknesses were grouped into the same formation group. The specific lithological parameters are shown in Table 2. The model used the Mohr-Coulomb material model, with a free boundary at the top and constraints applied to the other boundaries. The average depth of the working face coal seam is 500 m.

**Table 2:** Rock mechanics parameter.

Lithology	Density /kg/m^3^	Bulk modulus/MPa	Shear modulus/MPa	Cohesion /MPa	Tension /MPa	Internal friction angle /°
Fine sandstone	2400	8500	8990	6.54	4.63	31
Medium sandstone	2430	8130	9060	5.61	5.43	29
Mudstone	2130	5700	4100	3.47	3.15	25
Coal	1340	860	810	0.64	0.34	30
Mudstone	2000	3300	3700	3.43	2.91	25
Siltstone	2460	7700	8200	4.19	4.73	30

**Fig. 6:**
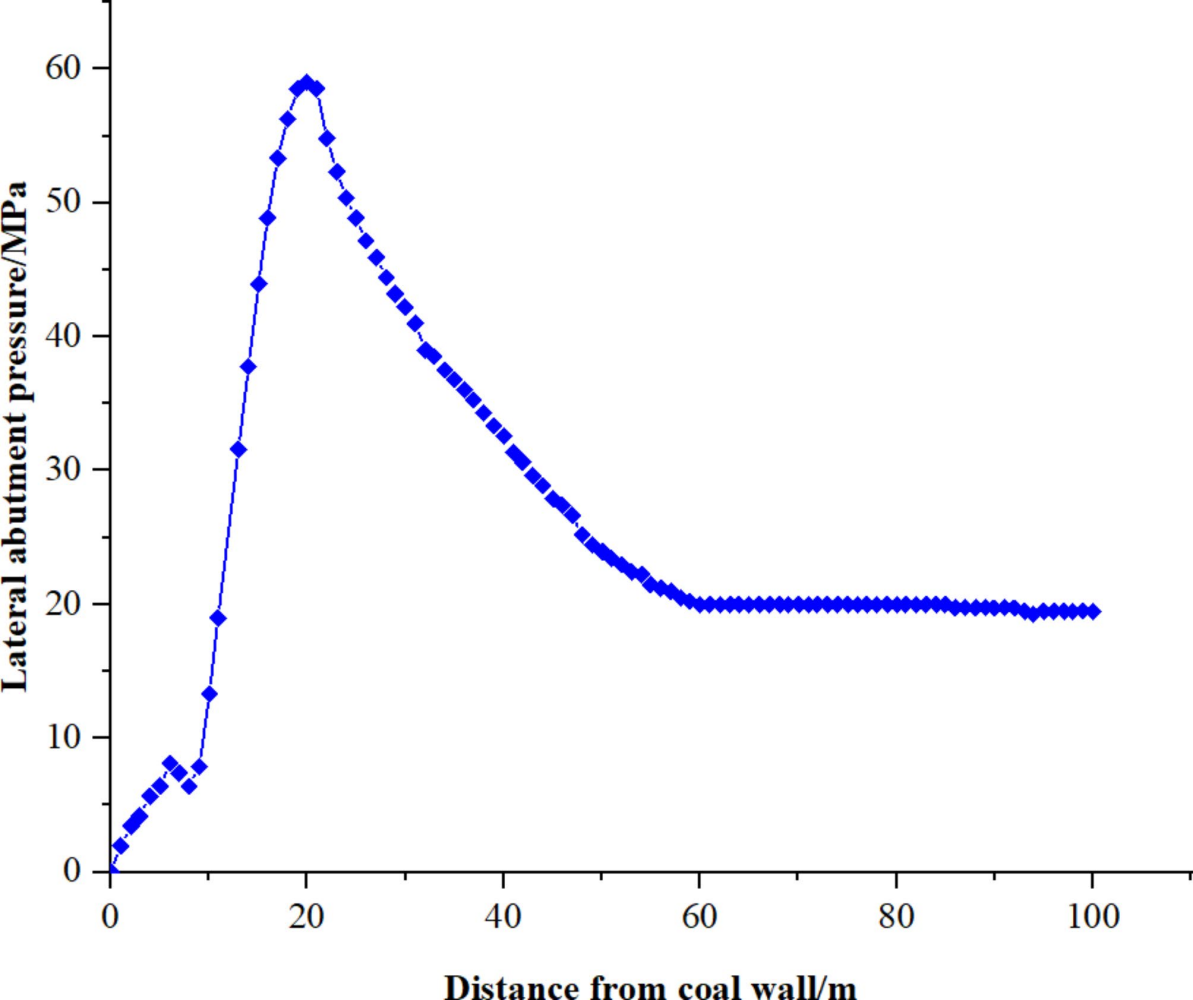
Curve of lateral support pressure distribution in working face.

According to Fig. 6, the abutment pressure in the lateral coal body shows a pattern of initial increase followed by a decrease with increasing distance from the coal wall. This pattern is categorized into three parts: low-stress zone, stress increase zone, and original rock stress zone. Notably, the extent of the low-stress zone aligns with the empirical measurements, estimated at about 8 m. Consequently, it is established that the low-stress zone in the lateral coal body extends to 8 m, suggesting that the width of the small coal pillar should not exceed this distance.

To ascertain the optimal size of the small coal pillar more precisely, numerical simulation methods were employed to analyze gob-side entry excavations with varying coal pillar widths. Accordingly, six simulation scenarios were devised, featuring coal pillars ranging in size from 3 m to 8 m at one-meter intervals.

**Fig. 7:**
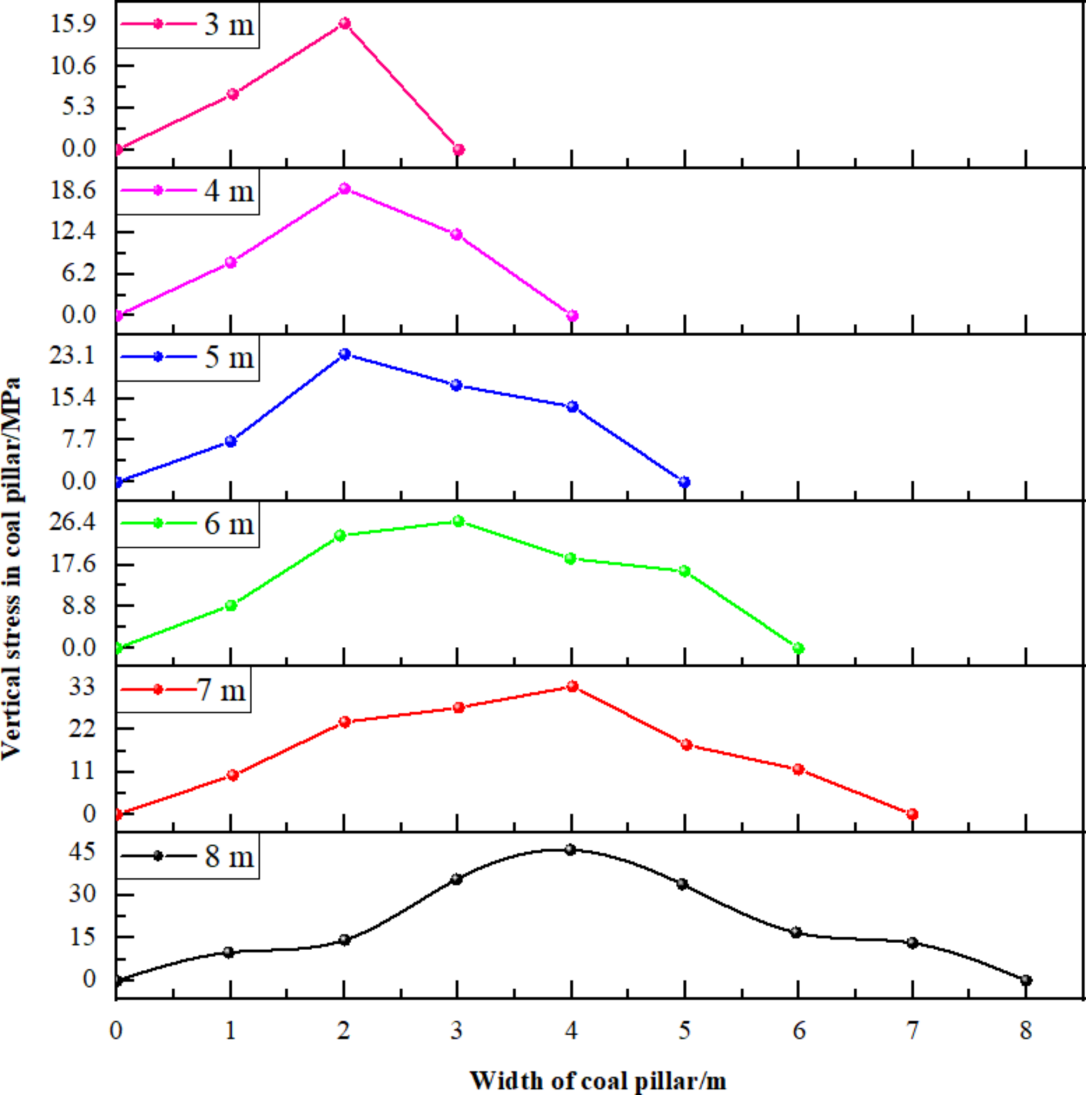
Vertical stress distribution of coal pillar with different width.

Figure 7 illustrates that the peak vertical stress increases nonlinearly with the size of the coal pillar. When the small coal pillar width is 3 m, its peak stress is 10.3 MPa, substantially below the stress of the original rock, suggesting that the 3 m coal pillar is compromised under abutment pressure and has significantly diminished bearing capacity. The internal stress of the 4 m coal pillar increases slightly, yet this rise is marginal, indicating that its bearing capacity improvement is also limited. When the coal pillar width is 5 m, the internal stress markedly escalates to an average abutment pressure of 22.1 MPa, indicating that the coal pillar at this width has a good bearing capacity. When the coal pillar width is between 6 m and 8 m, the stress concentration is high, and the peak stress continues to increase, with a stress concentration factor of 3.7. At these dimensions, the coal pillars are susceptible to instability and failure under high abutment pressure.

Considering all relevant factors, the optimal coal pillar width for gob-side entry excavation has been established at 5 m. Following this determination, it is crucial to set an appropriate entry excavation lag time to prevent roof subsidence and deformation of the surrounding rock, which can result from significant movements of the basic roof in the overlying working face.

### Study on the reasonable timing of gob-side entry excavation

**Fig. 8:**
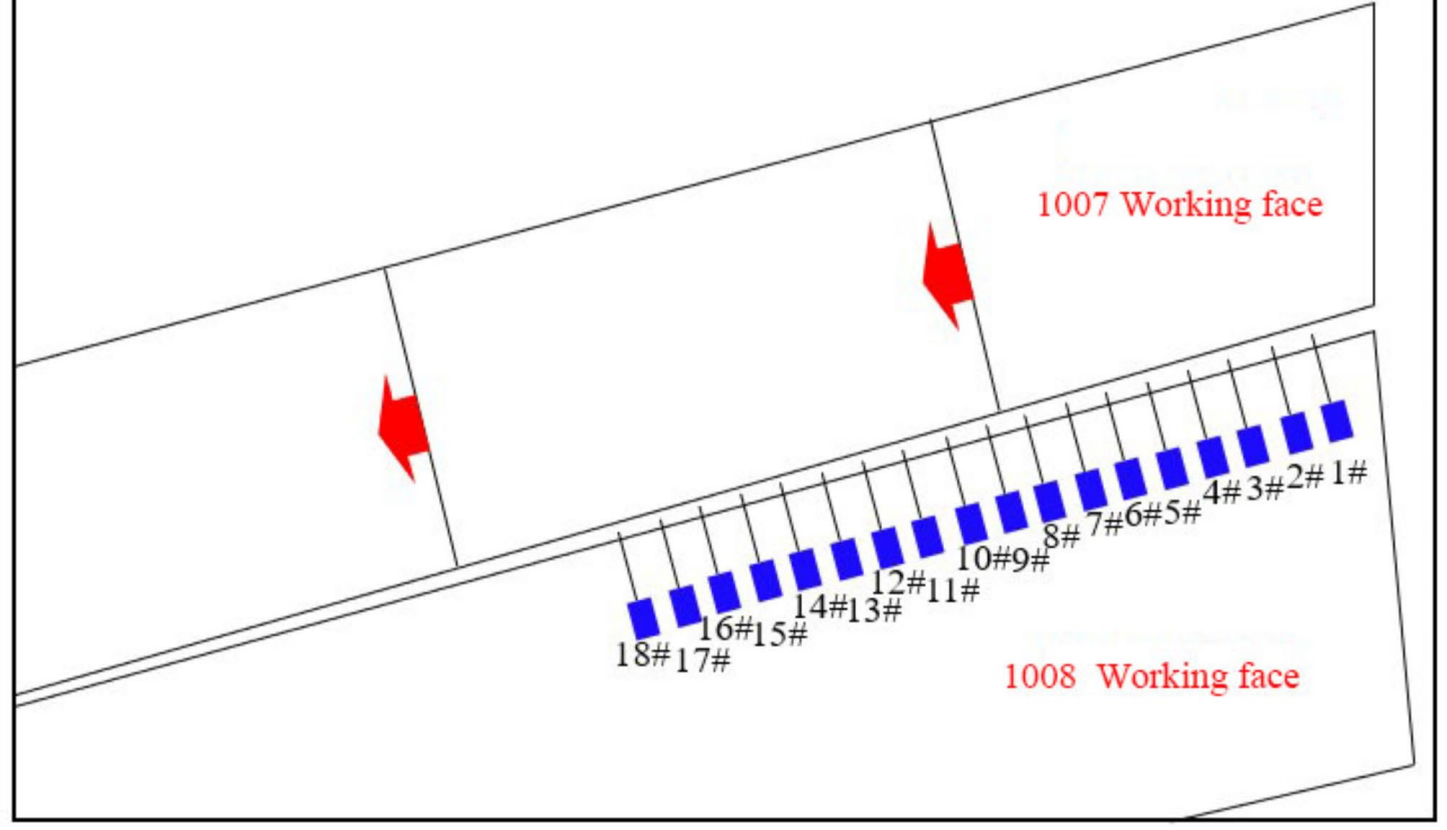
Survey station layout.

To alleviate the tension between mining and tunneling, a method was chosen to excavate the next entry behind the previous working face. During the retreat of the 1007 working face, deformation monitoring of the surrounding rock was performed at various locations along the gob-side entry of the 1008 working face. These varied locations corresponded to the lagging distances of the gob-side entry, illustrating the effects of different roof movement conditions on the deformation of the surrounding rock following the retreat of the 1007 working face. By analyzing the deformation of the surrounding rock at varying lagging distances of the gob-side entry, the stabilization time required for the basic roof after intense movement following the retreat of the upper working face can be determined, thereby establishing a reasonable timing for gob-side entry excavation. When the gob-side entry of the 1008 working face was lagging behind the 1007 working face, 18 surface displacement monitoring stations were set up to monitor the surface displacement of the roadway. The monitoring plan layout is shown in Fig. 8.

**Fig. 9:**
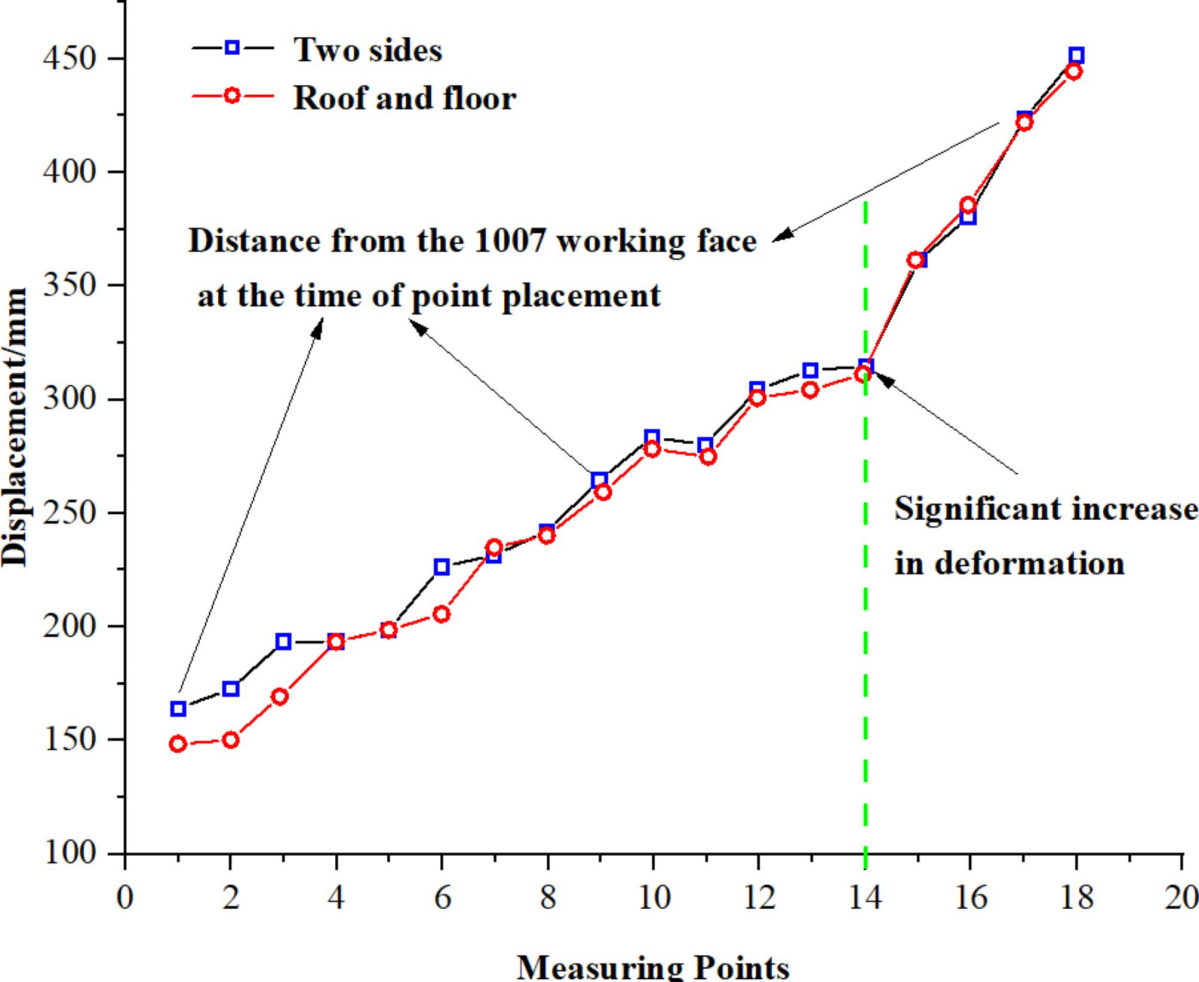
Each point set up within 30 days of accumulated deformation.

Given the varying observation durations at each measurement point, a clearer analysis of the roadway deformation pattern between the 1008 materials roadway heading and the 1007 working face at different lagging distances and times was conducted. This involved summarizing the cumulative deformation recorded within 30 days of establishing each measurement point. The results are depicted in Fig. 9.

When the lag distance between the gob-side entry excavation and the 1007 working face is under 159 m (equivalent to a lag time approximately 46 days), there is a marked increase in surrounding rock deformation. Additionally, a shorter the lag distance correlates with more severe deformation. This is primarily due to the intense movement of the 1007 working face roof impacting the surrounding rock at this proximity, causing significant deformation. Consequently, to mitigate conflicts between mining and tunneling, it is advised that the lag time for gob-side entry excavation be maintained at a minimum of 46 days.

## Numerical study on the deformation and failure characteristics of gob-side roadway surrounding rock

### Numerical simulation program

This section will analyze the effects of three key factors on surrounding rock stress and displacement: the burial depth of the coal seam, the cross-sectional size, and the mining thickness of the coal seam. The experimental plan comprises three distinct components: (1) Coal Seam Burial Depth Variation: This test maintains constant the roadway cross-sectional size, mining thickness, and width of the small coal pillar, while varying the burial depth of the coal seam. The stress and displacement distribution patterns of the surrounding rock at burial depths of 400 m, 500 m, 600 m, 700 m, and 800 m are simulated by adjusting the stress applied at the top of the model. (2) Cross-Sectional Size Variation: Based on existing dimensions of coal roadways in the mine, five simulation scenarios with varying cross-sectional sizes are established: 5.0 m × 4.6 m, 4.4 m × 4.2 m, 4.0 m × 3.6 m, 3.4 m × 2.8 m, and 2.8 m × 2.2 m. (3) Mining Thickness Variation: While maintaining constant mechanical parameters of the surrounding rock, various mining thicknesses are designed to reflect actual mining practices, with values set at 3.5 m, 4.0 m, 4.5 m, 5.0 m, and 5.5 m.

### Stress distribution pattern of surrounding rock

(1) Coal seam burial depth variation.

**Fig. 10:**
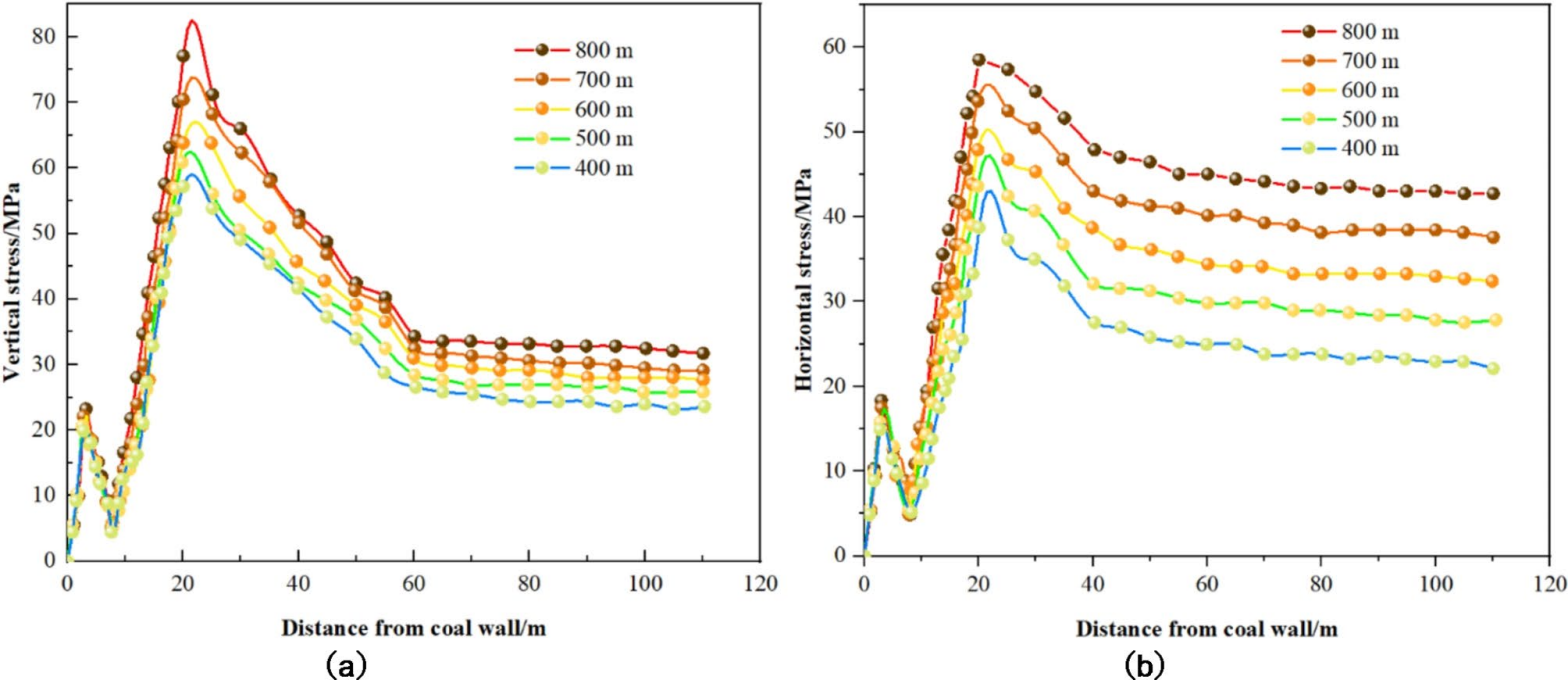
Stress distribution of solid coal in different burial depth. (**a**) Vertical stress. (**b**) Horizontal stress.

**Fig. 11:**
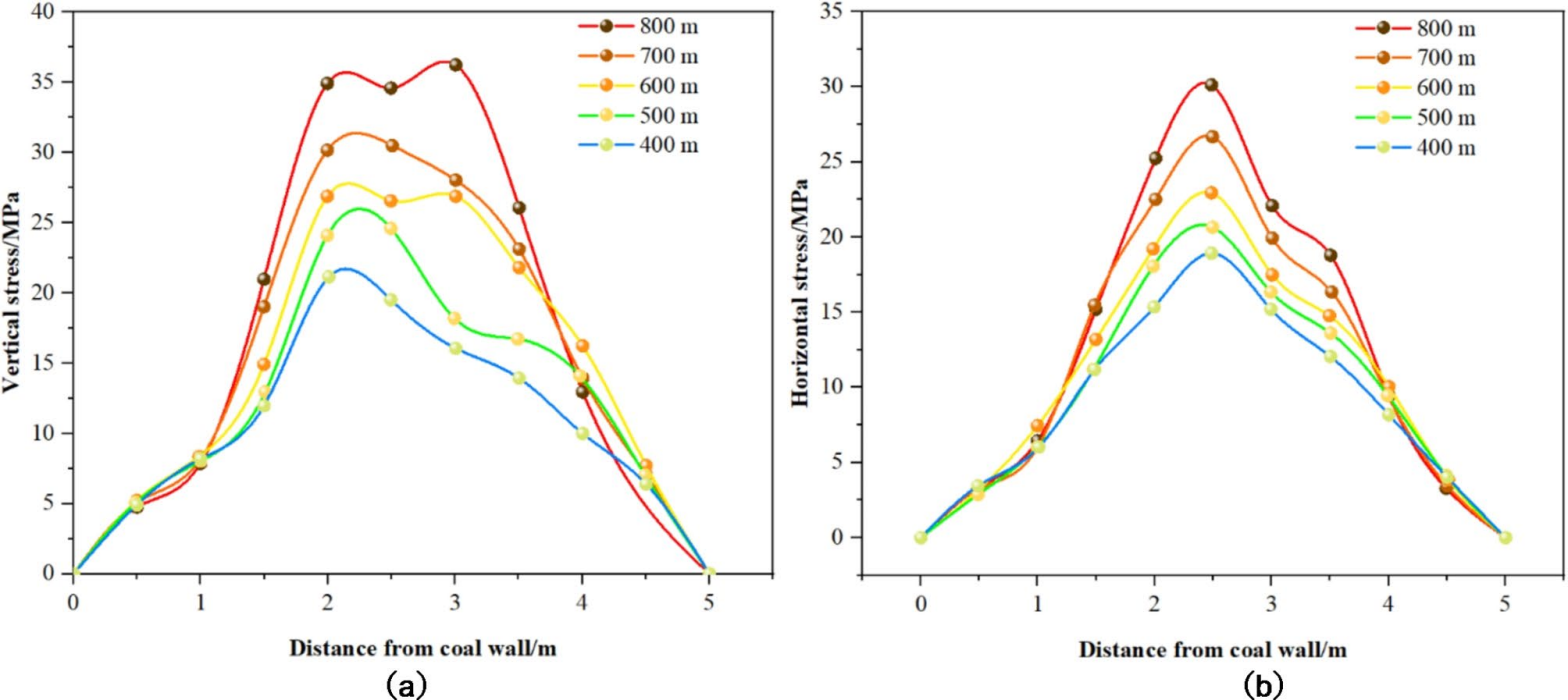
Distribution of stress of coal pillar at different depth of buried coal. (**a**) Vertical stress. (**b**) Horizontal stress.

Figure 10 illustrates the vertical and horizontal stress distribution curves for solid coal ribs at varying burial depths, demonstrating how these stresses change as burial depth increases. Each curve corresponds to a different condition, including depths of 400 m, 500 m, 600 m, 700 m, and 800 m. Similarly, Fig. 11 presents the stress distribution curves for coal pillar ribs under varying burial depths, showing changes in vertical and horizontal stresses with increased depth. Each curve also corresponds to specific burial depths such as 400 m, 500 m, 600 m, 700 m, and 800 m.

Observations from Figs. 10 and 11 include: ① Increase in Peak Stress with Burial Depth: The peak stress in the surrounding rock exhibits a positive correlation with increasing coal seam burial depth. Concurrently, the stress differential between the solid coal rib and the coal pillar rib widens; at a depth of 400 m, this difference is 35.7 MPa, escalating to 41.6 MPa at 800 m. ② Decrease in Coal Pillar Rib Shallow Stress: As burial depth increases, there is a noticeable decline in the shallow stress within the coal pillar rib, suggesting a progressive deterioration in the coal seam structure at shallow depths, which diminishes its load-bearing capacity. ③ Stress Distribution in Solid Coal Rib: The distribution of stress within the solid coal rib indicates minimal change in the peak stress position of the surrounding rock with increasing burial depth. From 400 m to 800 m, the peak stress position shifts slightly deeper.

(2) Cross-sectional size variation.

**Fig. 12:**
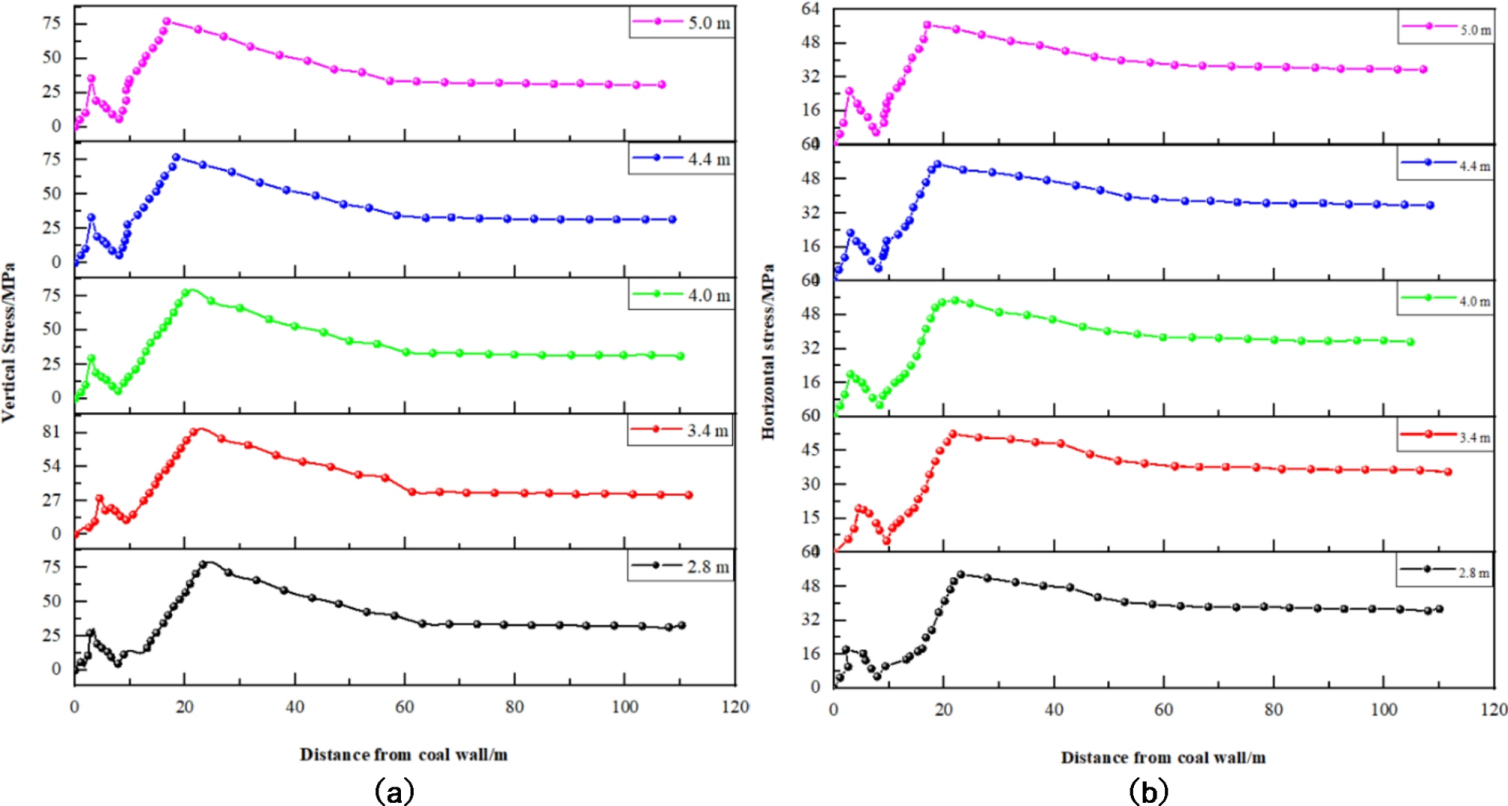
Curve of stress distribution of solid coal in different section sizes. (**a**) Vertical stress. (**b**) Horizontal stress.

**Fig. 13:**
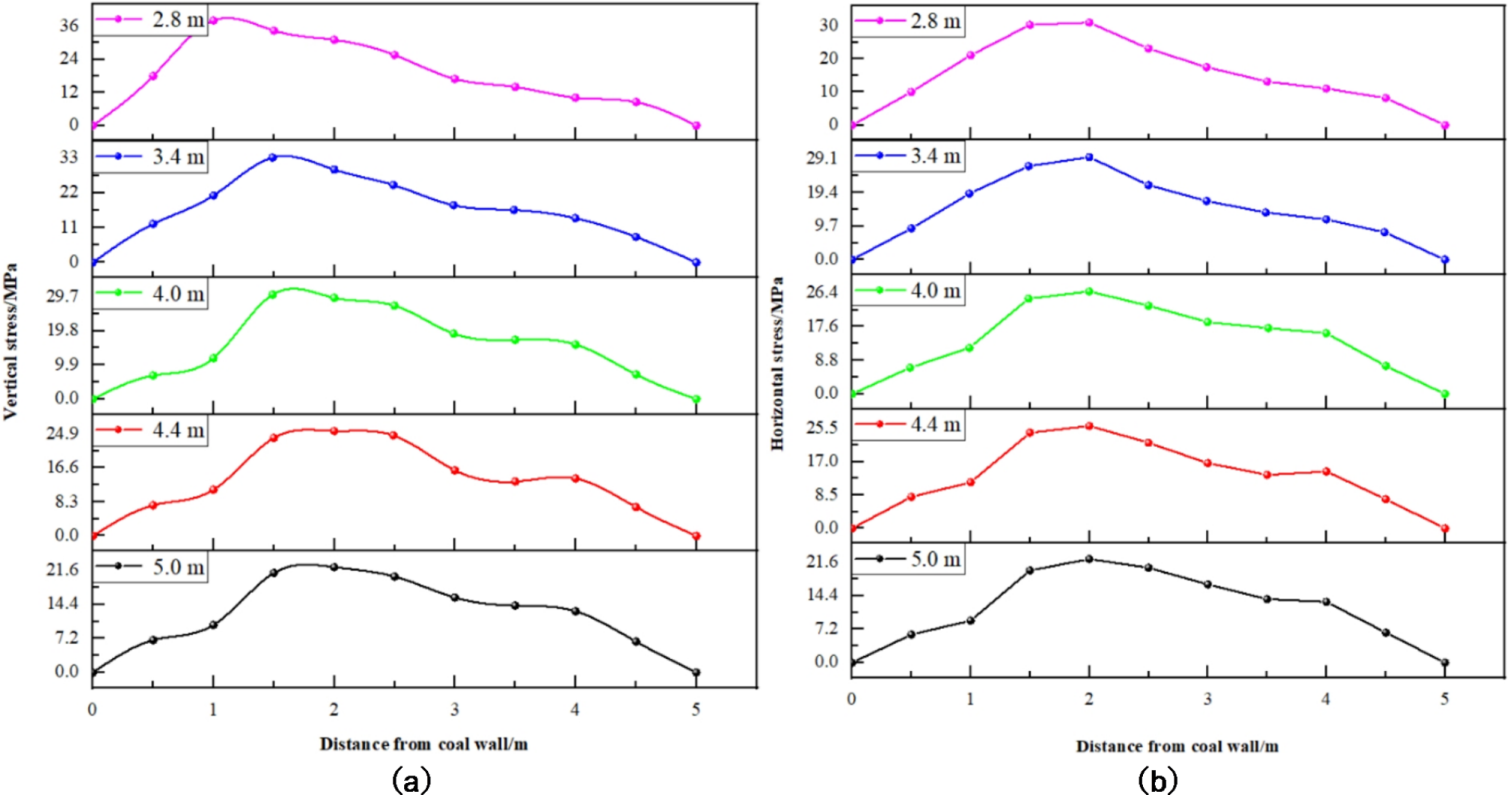
Curve of stress distribution of coal pillar with different section sizes. (**a**) Vertical stress. (**b**) Horizontal stress.

Based on the five designated schemes, numerical calculations were conducted, yielding the stress distribution curves presented in Figs. 12 and 13. Figure 12 illustrates the stress distribution curves of solid coal ribs along the gob-side roadway for varying cross-sectional sizes, while Fig. 13 depicts the stress distribution curve of the coal pillar rib.

Analysis from Figs. 12 and 13 yields the following insights: ① Stress Distribution in Solid Coal Rib (Fig. 12): While the overall pattern of stress distribution in the solid coal rib remains largely unchanged with increases in roadway cross-sectional size, the peak stress diminishes as the size expands. Notably, a larger cross-sectional size shifts the peak stress deeper into the roadway, suggesting a reduced bearing capacity in the roadway’s shallow sections. ② Vertical Stress Distribution in the Coal Pillar Rib (Fig. 13): The vertical stress distribution within the coal pillar rib exhibits notable variations with changes in cross-sectional size. Specifically, the stress distribution is triangular for roadway widths of 2.8 m and 3.4 m, and transitions to a trapezoidal shape for widths between 4.0 m and 5.0 m. ③ Influence of Cross-Sectional Size on Peak Vertical Stress Location in the Coal Pillar Rib: Increasing the cross-sectional size causes the peak vertical stress within the coal pillar to progressively move closer to the goaf. For instance, at a roadway width of 2.8 m, the peak stress is located 1 m from the coal wall, increasing to 1.5 m for widths of 3.4 m and 4.0 m, and reaching 2 m at widths of 4.4 m and 5.0 m.

(3) Mining thickness variation.

**Fig. 14:**
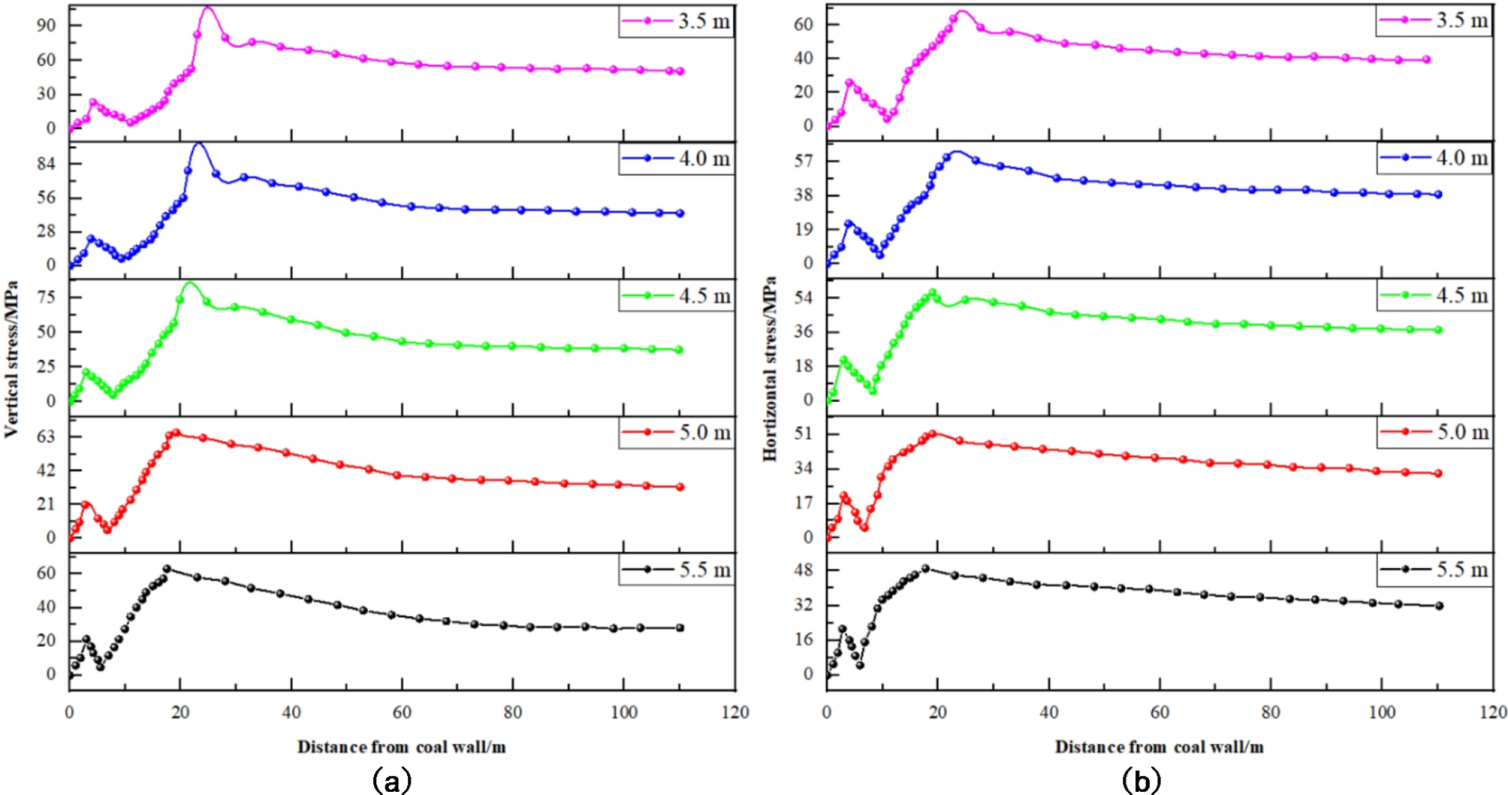
Stress distribution curve of solid coal in different mining thickness. (**a**) Vertical stress. (**b**) Horizontal stress.

**Fig. 15:**
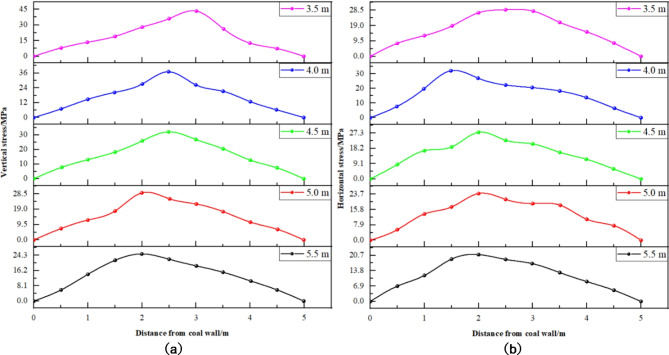
Stress distribution curve of coal pillar at different thickness of mining. (**a**) Vertical stress. (**b**) Horizontal stress.

Numerical simulations were conducted based on the five distinct mining thickness schemes, yielding the stress distribution patterns of the surrounding rock along the empty tunnel for each configuration, as illustrated in Figs. 14 and 15.

Insights derived from Figs. 14 and 15 are summarized as follows: ① Mining Thickness and Vertical Stress in Solid Coal Rib (Fig. 14): The vertical stress in the solid coal rib is significantly affected by changes in mining thickness. As the mining thickness increases from 3.5 m to 5.5 m, there is a notable reduction in peak vertical stress by 20 MPa. Similarly, the peak vertical stress in the coal pillar rib decreases by 18 MPa with increased mining thickness. ② Position of Peak Vertical Stress in Solid Coal Rib (Fig. 15): The position of peak vertical stress in the solid coal rib shifts closer to the coal wall with increasing mining thickness, moving from 3 m away at 3.5 m thickness to 1.5 m away at 5.5 m thickness. ③ Mining Thickness and Vertical Stress in Coal Pillar Rib (Fig. 15): As with the solid coal rib, increased mining thickness leads to a decrease in peak vertical stress within the coal pillar rib, with a less pronounced stress distribution pattern. ④ Peak Vertical Stress Position in Coal Pillar Rib (Fig. 15): The peak stress position in the coal pillar rib moves closer to the goaf with increasing mining thickness, suggesting a decrease in the coal pillar’s load-bearing capacity due to reduced stress concentration.

### Distribution pattern of surrounding rock displacement

In the gob-side roadway, the deformation of the surrounding rock is dominated by the coal pillar rib, so the deformation of the coal pillar is selected as an example to be analyzed in this section.

(1) Coal seam burial depth variation.

**Fig. 16:**
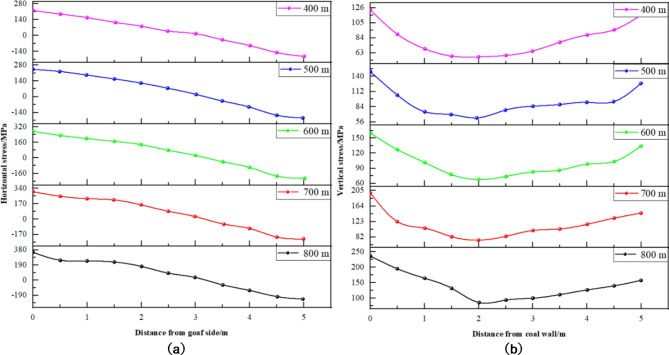
Displacement distribution curve of coal pillar when buried depth of different coal seams. (**a**) Horizontal displacement. (**b**) Vertical displacement.

Insights from Fig. 16 are as follows: ① Displacement Trends with Increasing Coal Seam Burial Depth: With deeper coal seam burial, both horizontal and vertical displacements of the coal pillar rib along the gob-side roadway increase. Deformation is notably more pronounced on the goaf side than on the roadway side, with horizontal displacement reaching 221 mm and vertical displacement 123 mm at a burial depth of 400 m. ② Impact of Burial Depth on Displacement Distribution Patterns: Variations in burial depth appear to have minimal impact on the displacement distribution patterns within the coal pillar rib. The displacement, both horizontal and vertical, tends to be symmetrically concentrated around the central area of the coal pillar, with slightly greater displacement on the goaf side compared to the roadway side.

(2) Cross-sectional size variation.

**Fig. 17:**
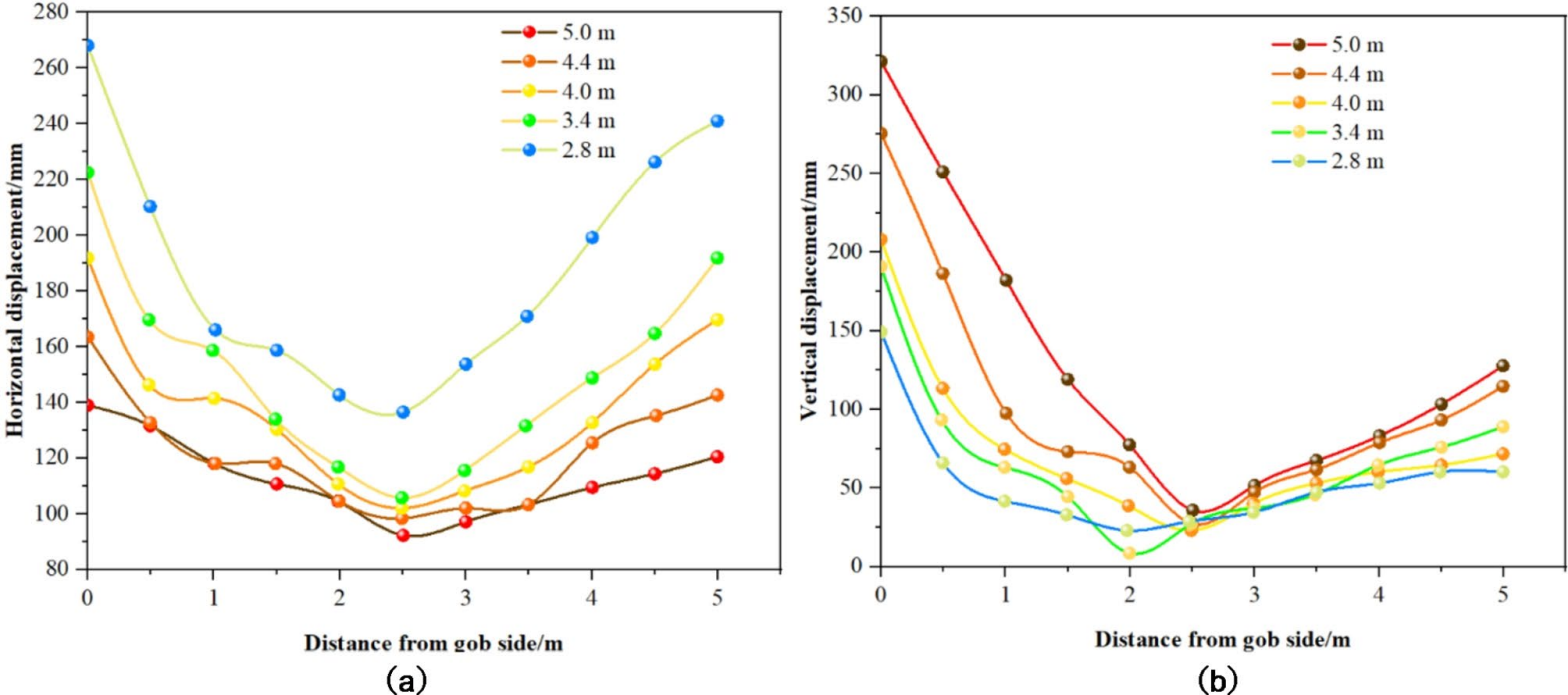
Displacement distribution curve of coal pillar with different section sizes. (**a**) Horizontal displacement. (**b**) Vertical displacement.

Insights derived from Fig. 17 include: ① Impact of Cross-Sectional Size on Displacement in Coal Pillar Rib: An increase in the cross-sectional size of the roadway leads to a corresponding increase in both horizontal and vertical displacements of the coal pillar rib. Displacements are notably lower in the middle of the coal pillar compared to the sides, with greater displacement generally observed on the goaf side than the roadway side. Expanding the roadway’s width and height enlarges the roof span, shifting the load towards the coal pillar’s sides and diminishing its load-bearing capacity. ② Displacement Variations by Roadway Width: At a roadway width of 2.8 m, horizontal and vertical displacements measured 247 mm and 149 mm, respectively. Increasing the width to 5.0 m results in horizontal and vertical displacements of 478 mm and 321 mm, respectively. ③ Magnitude of Displacement Increase: The horizontal displacement increased by 231 mm (from 247 mm to 478 mm), representing a 93.5% rise. Vertical displacement increased by 172 mm (from 149 mm to 321 mm), marking a 115.4% rise.

(3) Mining thickness variation.

**Fig. 18:**
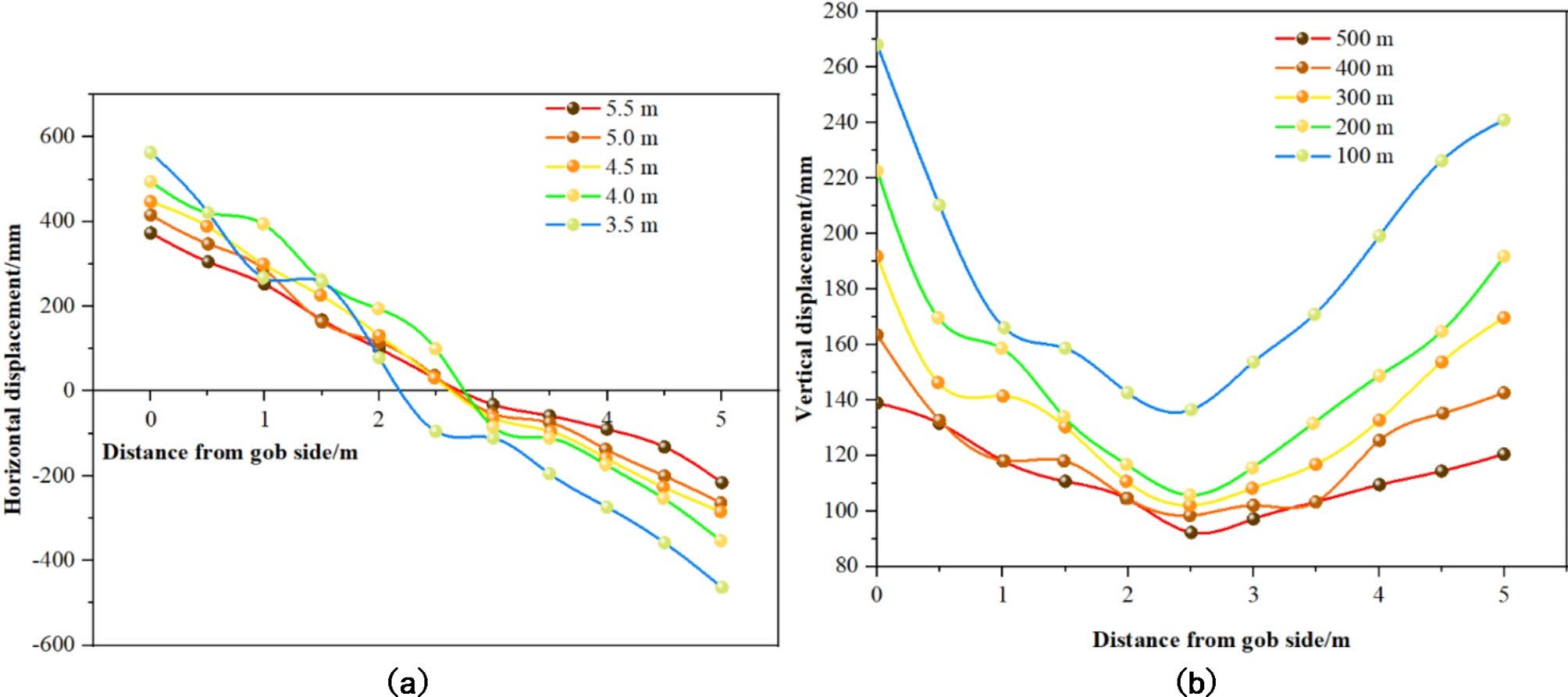
Displacement distribution curve of coal pillar when mining thickness of different coal seams. (**a**) Horizontal displacement. (**b**) Vertical displacement.

Insights from Fig. 18 are summarized as follows: ① Impact of Mining Thickness on Coal Pillar Rib Displacement: With an increase in mining thickness, there is a gradual reduction in both horizontal and vertical displacements of the coal pillar rib. Specifically, at a mining thickness of 3.5 m, the coal pillar rib experiences a maximum horizontal displacement of 562 mm and a maximum vertical displacement of 268 mm. When the mining thickness is enhanced to 5.5 m, the horizontal displacement diminishes to 374 mm, and the vertical displacement reduces to 139 mm. ② Displacement Distribution Pattern as Mining Thickness Increases: Despite changes in mining thickness, the displacement distribution pattern of horizontal and vertical movements within the coal pillar rib remains consistent. Notably, between 2 m and 3 m from the goaf, both displacement types are considerably lower, suggesting that this segment serves as the stable load-bearing zone of the coal pillar.

## Study on surrounding rock control measures for gob-side entry excavation

### Original support scheme and its measured analysis

#### Observation program

In the 1007 materials roadway, a laser rangefinder was positioned 178 m from the crosscut to monitor surface displacement of the surrounding rock. Furthermore, two sets of anchor bolt working resistance monitoring stations were established on either side of the roadway. Considering that the materials roadway is flanked by solid coal and a coal pillar designated for gob-side entry excavation, anchor bolt load cells were installed on both sides to assess structural integrity.

#### Analysis of anchor bolt working resistance monitoring results

**Fig. 19:**
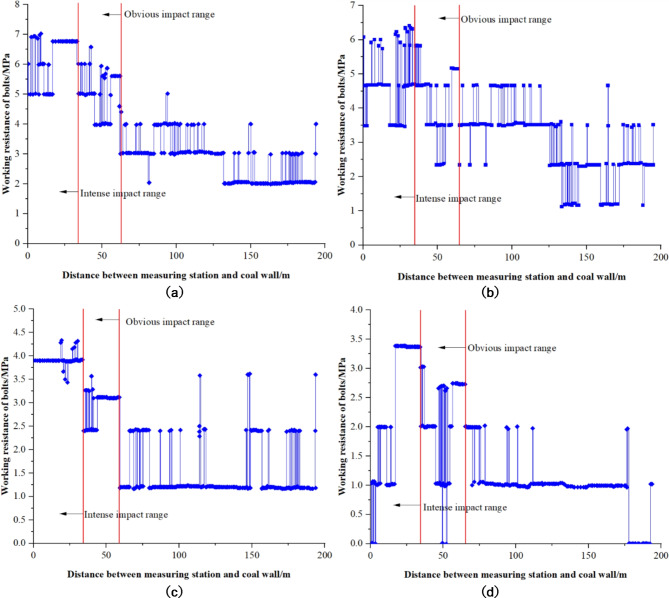
Measured results of working resistance of anchor bolts at various stations. (**a)** Anchor Bolt Load Cell #1. (**b**) Anchor Bolt Load Cell #2. (**c**) Anchor Bolt Load Cell #3. (**d**) Anchor Bolt Load Cell #4.

In 1007 materials roadway, four measuring stations were established, each equipped with two anchor bar dynamometers. The results of the anchor work resistance measurements at each station are presented in Fig. 19.

**Fig. 20:**
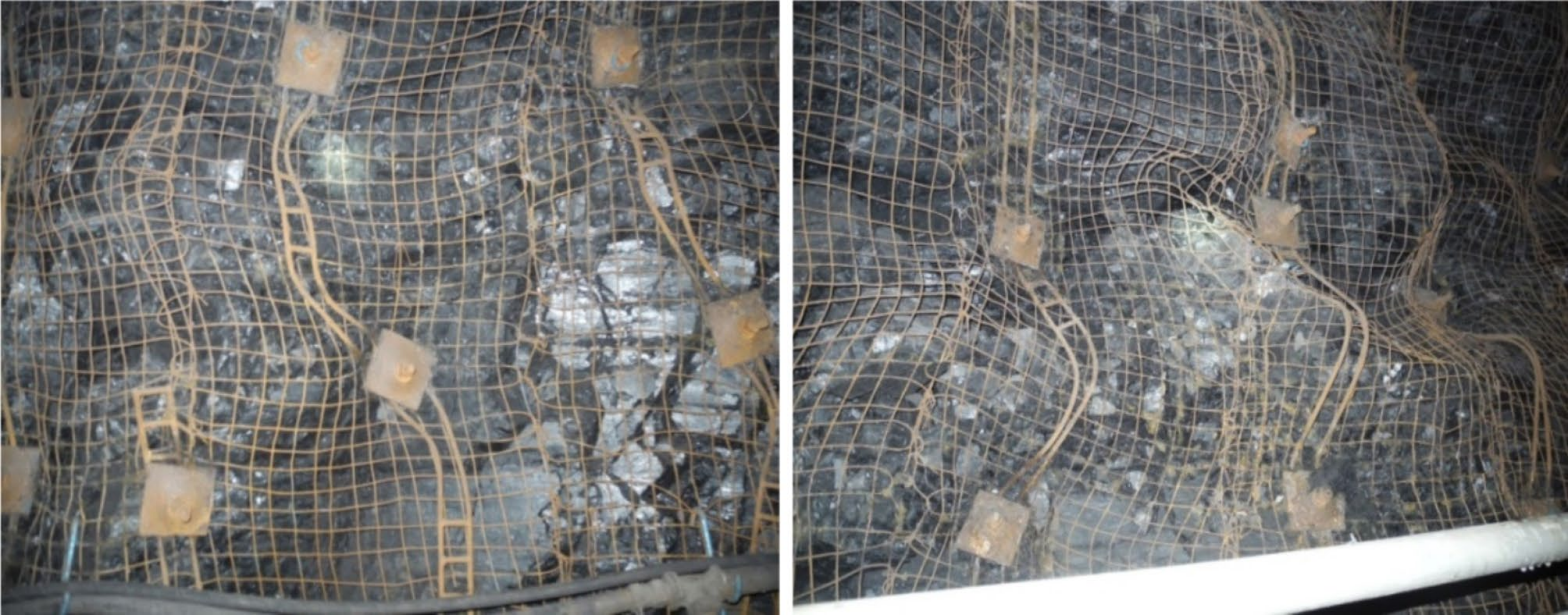
Bending deformation of steel bar ladder.

From the anchor working resistance measurements, it is evident that:

(1) Anchor Bolt Load Cell #1 sustained its initial tension for an extended period post-installation. This longevity in tension primarily resulted from the anchor bolts’ relatively low pretension, hindering their ability to offer effective and timely support amidst minor deformations of the surrounding rock.

(2) Monitoring data indicate that the installation pre-tension of the anchor bolts was relatively low, mainly ranging between 1 and 4 MPa, with an average of 3 MPa. This low level of pre-tension hindered the bolts’ ability to effectively and quickly counteract surrounding rock deformation, consequently limiting their working resistance.

(3) Steel reinforcement ladders were utilized for sidewall support in the mining roadway. Despite their deployment, these ladders were found to be deficient in strength and stiffness. Field observations (Fig. 20) revealed numerous reinforcement ladders that were twisted, deformed, or broken, compromising the structural integrity and support effectiveness of the roadway. The deformation or breakage of these ladders impeded their ability to effectively transmit the pretension from the anchor bolts, thereby substantially diminishing or nullifying the bolts’ working resistance.

(4) Analysis of the trend of in anchor working resistance at varying distances from the working face enables the identification of zones with significant and severe impacts due to over-support pressure. For instance, for anchor 1#, as the working face approaches within 61.5 m of the station, there is a noticeable increase in working resistance. This increase becomes more pronounced when the working face is 33.7 m from the station. Consequently, it can be inferred that the peripheral rock around Station #1, located 194 m from the cutting eye, experiences significant effects from the over-support pressure within 61.5 m of the working face, with severe impacts occurring within 33.7 m. This methodology allows for the determination of support pressure impacts at each station, as detailed in Table 3.

**Table 3:** The influence range of support pressure determined according to the analysis of bolt working resistance.

Station number	1#		2#		Average value
Bolt force meter No.	1#	2#	3#	4#	—
Significant Influence Range of Abutment Pressure /m	61.5	62	58	63	61
Significant Influence Range of Advance Abutment Pressure/m	33.7	31.8	34.2	32.3	33

As indicated in Table 3, the material roadway exhibits significant impact from the working face overburden pressure within 61 m ahead of the working face, with severe effects observed within 33 m.

#### Analysis of surface displacement observations

**Fig. 21:**
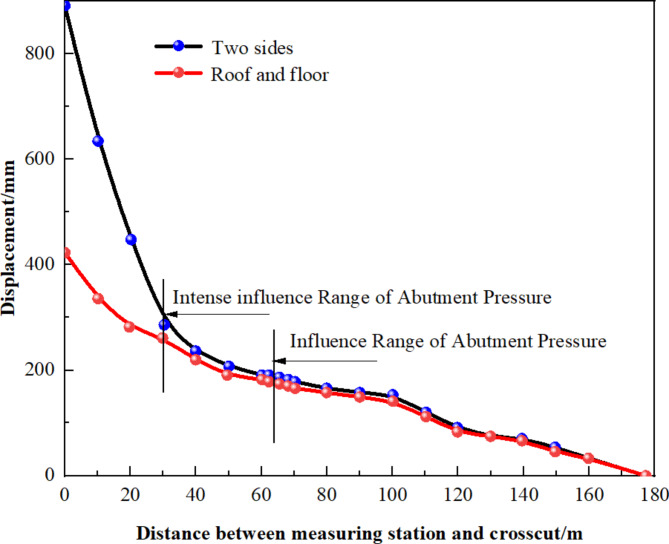
Measured results of surface displacement of clockwise surrounding rock.

In the 1007 material roadway, a laser rangefinder was positioned 178 m from the crosscut to monitor the surface displacement of the surrounding rock. The measuring station was capable of simultaneously tracking displacement of the roof, floor, and both sides. The results of these measurements are presented in Fig. 21.

Key findings from the deformation monitoring of the surrounding rock in the gob-side roadway of the 1007 working face include: (1) Displacement of Roof, Floor, and Ribs: Over the 178-m stretch from the crosscut to the measuring point, the roof and floor converged by 420 mm, yielding a convergence rate of 9.55%. Rib convergence totaled 892 mm, with a rate of 21.24%. Major factors contributing to significant roadway deformation include: ① Low Bolt Pretension and Working Resistance: The insufficient pretension and working resistance of the anchor bolts limit their effectiveness in providing active and timely support to the surrounding rock. ② Large Section Roadway and High Extraction Height: The large dimensions of the roadway, coupled with a full-section extraction height of 5.5 m at the working face, amplify the range and intensity of advance abutment pressure, resulting in pronounced deformation and failure of the surrounding rock.

**Table 4:** Statistical table of surrounding rock displacement in laneway.

Items		Surface displacement of material roadway
Total displacement of surrounding rock in the roadway /mm	Roof and floor	420
	Wall	892
Approach rate of roadway surrounding rock /%	Roof and floor	9.55
	Wall	21.24

(2) As detailed in Table 4, convergence within the material roadway on the gob side manifested as 420 mm between roof and floor, and a more pronounced 892 mm between the two ribs. This significant deformation in the gob-side roadway stemmed from mining-induced disturbances from the upper working face, leading to extensive and severe fracturing of the rock mass, thereby compromising structural stability. Further substantial deformations of the surrounding rock were exacerbated by the mining activities at the 1007 working face. Moreover, these disturbances had a heightened impact on the coal body along the two ribs, rendering it more susceptible to breakage. As a result, the convergence observed between the two ribs was notably greater than that between the roof and floor.

(3) Until the working face advanced within 65 m of the measuring station, convergence measurements between the roof, floor, and ribs in the material roadway exhibited minimal fluctuation, maintaining relative stability. However, as the working face neared within 65 m, notable increases in convergence both between the roof and floor and between the ribs commenced, with the convergence rate of the surrounding rock gradually accelerating. As the working face approached closer, at a distance of 32 m from the station, convergence sharply intensified, indicating significant deformation. This marks a critical impact zone, where mining activities substantially compromise the structural stability and integrity of the materials roadway.

Although significant deformation occurred in the surrounding rock of the gob-side roadway, it remained largely within a controllable range, primarily confined to the advance abutment pressure zone. To enhance support effectiveness, the following recommendations are proposed: Increase bolt pretension to more effectively engage and stabilize the surrounding rock; implement W steel strips with greater strength and rigidity to bolster support capacity; and strengthen support within the advance abutment pressure zone to mitigate surrounding rock deformation. Additionally, reinforce the support ahead of the materials roadway to ensure safety and stability during the retreat of the working face.

### Optimization of bolt support parameters for gob-side entry excavation

#### Principles for controlling surrounding rock stability in gob-side roadways

Based on the deformation and failure characteristics of the surrounding rock in the gob-side roadway of the 1007 working face, the following control principles are proposed:

(1) Improve the pretension of bolts and cables: Inadequate pretension can impede the timely and effective prevention of surrounding rock deformation, and insufficient working resistance of bolts and cables is particularly evident when the surrounding rock is weak and the roadway is under significant mining influence. Measurements indicate that the pretension of bolts and cables is generally low, contributing to the inadequate support performance. It is recommended to adopt practical measures to significantly enhance the pretension of bolts and cables, such as utilizing friction-reducing washers, employing high-torque drilling rigs, or supplementing with high-torque wrenches.

(2) Implement high-strength W steel belt. Currently, the pretension from the roadway bolt does not effectively transfer to the surrounding rock, diminishing the support provided. It is crucial to select support components that match the bolt rod to ensure that the anchoring force is efficiently transmitted to the roadway’s surrounding rock, enhancing the overall stiffness and integrity of the bolt assembly, and thereby improving the support effectiveness. High-strength W steel belts, compatible with the load-bearing capacity of individual bolts (or cables) and the extensive deformation of the surrounding rock, are recommended for this purpose.

(3) Reinforce weak sections of the roadway: The overall stability of the roadway is predominantly influenced by its weakest sections, where initial deformations typically emerge and subsequently propagate throughout the surrounding rock. These weak points often precipitate extensive roadway damage. Therefore, reinforcing these critical areas is crucial to enhancing the overall stability of the roadway’s surrounding rock structure.

#### Specific control measures for surrounding rock in gob-side roadway

Following the analysis of pressure monitoring issues in the gob-side roadway, support parameters for small coal pillar gob-side entry were optimized as follows: (1) Roof Bolt Parameters: High-strength prestressed metal bolts are employed in the roof. Spacing is set at 720 × 800 mm with six bolts per row. Bolts have a diameter of 20 mm and a length of 2400 mm, with pretension ranging from 5 to 7 tons and an installation torque of at least 200 N∙m. Surface support combines welded steel mesh and high-strength W steel strips. (2) Rib Bolt Parameters: Both ribs are supported by high-strength prestressed metal bolts, spaced at 800 × 800 mm with ten bolts per row. These bolts share the same diameter and length as the roof bolts, and the pretension and torque requirements are identical. Surface support on the production rib incorporates diamond-shaped metal mesh alongside high-strength W steel strips, while the coal pillar rib uses welded steel mesh with high-strength W steel strips. (3) Roof Cable Bolt Parameters: Steel strand cable bolts range from 6000 to 7000 mm in length, designed to penetrate the top coal layer, with a standard length of 6000 mm for normal sections. Cable bolts must maintain an installation stress of no less than 15 tons and feature a diameter of 21.8 mm to enhance load-bearing capacity. They are arranged at a spacing of 2160 × 2000 mm with two cables per row.

## Case study

**Fig. 22:**
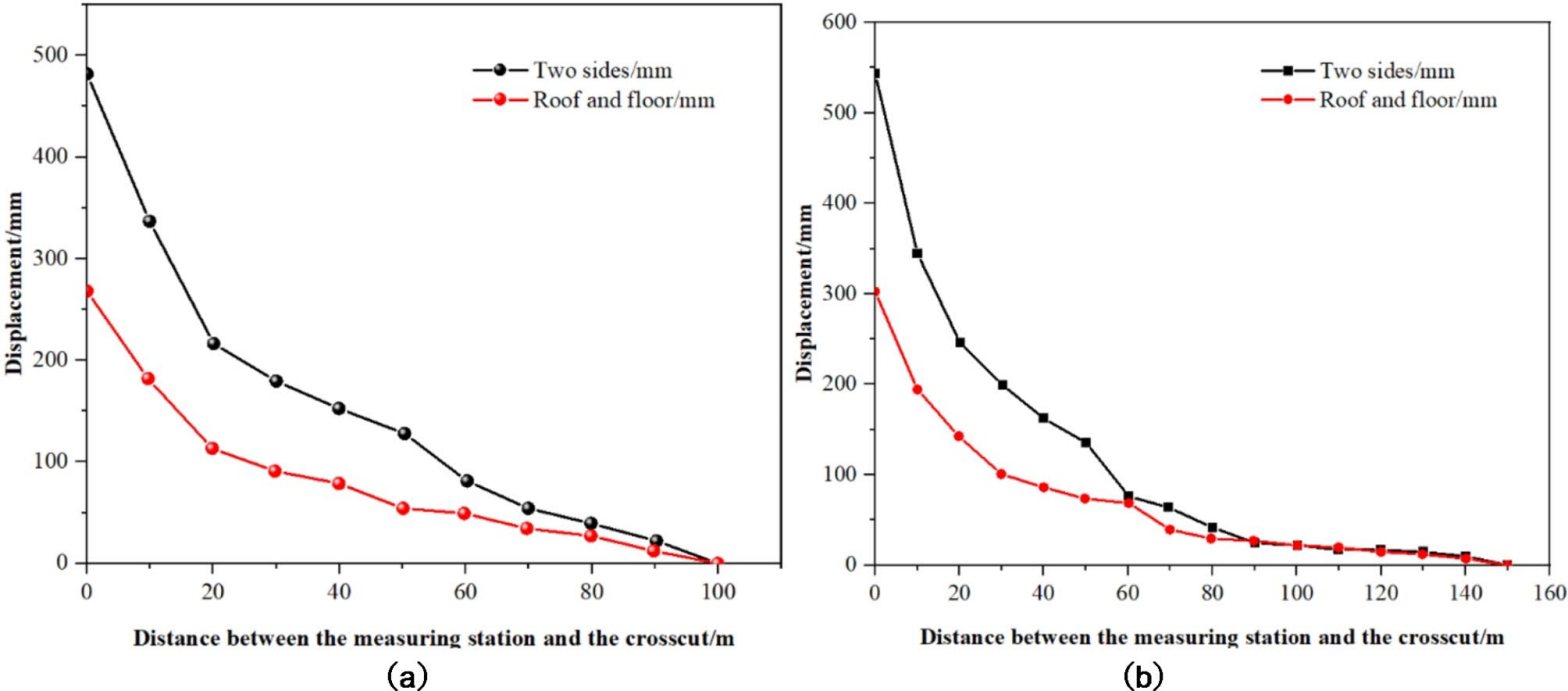
Measured results of deformation of roadway surrounding rock. (**a**) Deformation of surrounding rock at station #1. (**b**) Deformation of surrounding rock at station #2.

In the material roadway (gob-side roadway) of the 1008 working face, two monitoring stations were established at distances of 100 m and 150 m to monitor changes in surface displacement of the surrounding rock as the working face progressed. Figure 22 illustrates the trend of displacement changes, and Table 5 summarizes the convergence of the surrounding rock using the original and optimized support schemes.

**Table 5:** The statistical table of surrounding rock convergence.

Monitoring stations		Monitoring distance /m	Total displacement of the wall of the roadway /mm	Total displacement of the roof and floor of the roadway /mm
The original support schemes	1	178	892	420
The optimized support schemes	1	100	483	267
	2	150	543	303

Based on Fig. 22; Table 5, deformation observations at the 1# measuring station indicate minimal rock movement within 60 m from the crosscut, with a slow deformation rate. However, between 60 m and 22 m from the crosscut, the rate of deformation began to accelerate to an average of 3.2 mm/m. Closer to the crosscut, within 22 m, the rate further increased to 12.5 mm/m. The cumulative convergence between the roof and floor reached 267 mm, corresponding to a 6.35% convergence rate, while rib convergence totaled 483 mm at a rate of 10.98%. At the 2# station, before reaching 90 m from the crosscut, deformation remained negligible. From 91 to 58 m, deformation gradually increased to 1.8 mm/m. As the face approached between 58 and 25 m, the rate climbed to 2.7 mm/m, and within 25 m of the crosscut, it spiked to 12.6 mm/m. Here, the total roof-floor convergence was 303 mm with a 7.21% rate, and rib convergence was 543 mm at a 12.34% rate. When compared to the materials roadway of the 1007 working face, there was a significant reduction in overall deformation, suggesting that the implemented control measures and optimized support parameters are effective, demonstrating enhanced support performance.

## Conclusions

(1) Field measurements and numerical simulations demonstrate that the optimal advance abutment pressure influence extends up to 57.3 m ahead of the working face, with peak pressures at 23.6 m and an effective low-stress zone starting around 8 m from the coal wall. Simulations further suggest that a 5-meter coal pillar is ideal for balancing load-bearing capacity and minimizing stress-induced failures.

(2) Field observations from the 1008 working face show that the new support scheme significantly reduces deformation compared to the 1007 face, indicating enhanced adaptability and control of surrounding rock conditions in thick coal seams. This supports the effectiveness of the strategy of increasing bolt pretension, using high-strength steel strips, and reinforcing weak points.

(3) Continuous monitoring has proven crucial for assessing the long-term performance of the optimized support system and informing adjustments to the support strategy and coal pillar dimensions to ensure ongoing stability and meet mining requirements.

## Data Availability

All data generated or analysed during this study are included in this published article.

## References

[1] Wu, Z. G. & Wang, Z. L. Shi. Narrow coal pillar stress calculation of God Side Entry Retaining. *Coal Min. Technol.* **24**, 26–29 (2019).

[2] Cheng, L. X. et al. Deformation and failure characteristics and control technology of surrounding rocks in deeply gob-side entry driving. *J. Min. Saf. Eng.* **38**, 227–236 (2021).

[3] Lu, S. F. et al. Deformation and Control Measures of Narrow Coal Pillar in Gob-Side Entry. *Min. Res. Dev.* **40**, 28–31 (2020).

[4] Wang, Q. et al. Comparative study of Model tests on automatically formed Roadway and Gob-Side Entry driving in Deep Coal Mines. *INT. J. MIN. SCI. TECHNO*. **31**, 591–601 (2021).

[5] Liu, H. et al. Research on roof damage mechanism and Control Technology of Gob - Side Entry Retaining under Close Distance Gob. *ENG. FAIL. ANAL.* **138**, 106331 (2022).

[6] Wang, Q. et al. Study of a No-Pillar mining technique with automatically formed gob-side entry retaining for Longwall Mining in Coal Mines. *INT. J. ROCK. MECH. MIN.* **110**, 1–8 (2018).

[7] Niu, T., Wang, F. & Wang, W. Key factors and Control Technology of Energy-Gathered Instability of Coal Pillar. *J. Min. Strata Control Eng.* **2**, 23017 (2022).

[8] Dong, H. Ground Control of Narrow Coal Pillar in Gob Side Entry driving with fully mechanized top coal Caving Mining in Extra-thick Coal Seam. *J. Min. Strata Control Eng.* **3**, 33017 (2021).

[9] Qin, Z., Gao, J. & Zheng, X. Bearing characteristics and Rock Burst mechanism of wide coal pillar with defect along Gob. *J. Min. Strata Control Eng.* **2**, 23021 (2023).

[10] Tian, C. Y. et al. Narrow coal pillar width and surrounding rock control of gob-side entry driving in 6 m high-cutting working face. *Coal Eng.* **53**, 39–44 (2021).

[11] Chen, X. X., Zhang, T. & Wang, Y. L. Study on influencing factors of surrounding rock deformation and asymmetric support technology for roadway driving along goaf with narrow coal pillars. *J. Henan Polytechnic University(Natural Science)*. **40**, 24–33 (2021).

[12] Dong, H. X. Ground control of narrow coal pillar in gob side entry driving with fully mechanized top coal caving mining in extra-thick coal seam. *J. Min. Strata Control Eng.* **3**, 32–42 (2021).

[13] Chen, Z. W. & Zhang, Y. D. Research and application of coordinated control technology for surrounding rock stability of gob–side entry driving with narrow coal pillar. *Min. Saf. Environ. Prot.* **50**, 65–70 (2023).

[14] Kang, H., Wu, L., Gao, F., Lv, H. & Li, J. Field Study on the Load Transfer Mechanics Associated with Longwall Coal Retreat Mining. *INT. J. ROCK. MECH. MIN.* **124**, 104141 (2019).

[15] Zhu, H., Wen, Z., Xu, L. & He, F. Key Technology of Gob-Side Entry retained by roof cutting without coal Pillar for hard main roof: a typical case study. *J. CENT. SOUTH. UNIV.* **30**, 4097–4121 (2023).

[16] Wang, M. et al. Stability Control of Overburden and Coal Pillars in the gob-side entry under dynamic pressure. *INT. J. ROCK. MECH. MIN.* **170**, 105490 (2023).

[17] Zhang, L. et al. Evolutionary Law and Regulatory Technology of roof Migration on Gob-Side Entry Retaining. *SCI. REP-UK* **14**, 5581 (2024). 10.1038/s41598-024-56108-z PMC10917779 38448473

[18] Fu, Q. et al. Mechanism and surrounding Rock Control of Gob-Side entry formation passing through normal Fault: a Case Study. *ENVIRON. EARTH SCI.* **82**, 603 (2023).

[19] Shan, C., Shang, H. & Zhang, Q. Width and bearing strength check of ‘’Two hard’’ coal Pillar in fully mechanized caving face. *J. Min. Strata Control Eng.* **6**, 23022 (2024).

[20] Hao, X., Han, G. & Xie, J. Rock Burst mechanism of Roadway Excavation along Goaf with Small Coal Pillar in Ordos Mining Area. *J. Min. Strata Control Eng.* **5**, 23017 (2023).

[21] Jia, C. & Hu, C. Instability mechanism and Control Technology of Longwall Entries Driving along the Gob in a thick coal Seam. *J. Min. Strata Control Eng.* **2**, 43535 (2020).

[22] Yang, H. Pu. Research and discussion on gob–side entry driving technology with narrow coal pillar in thick coal seam. *Coal Sci. Technol.* **49**, 67–70 (2021).

[23] Gao, X. J., Zhang, H. Q., Zhang, Z. & Li, S. J. Hai. Research on Coal Pillar size of gob–side entry driving and surrounding Rock Control Technology in Extra-thick Coal Seam. *Coal Technol.* **41**, 36–40 (2022).

[24] Huang, W. P. et al. Arrangement of double entry driving with a narrow coal pillar in the middle and stability control technology of surrounding rock. *Chin. J. Rock Mechan. Eng.* **42**, 617–629 (2023).

[25] Salmi, E. F., Karakus, M. & Nazem, M. Assessing the effects of Rock Mass Gradual Deterioration on the Long-Term Stability of Abandoned Mine workings and the mechanisms of Post-mining Subsidence - A Case Study of Castle Fields Mine. *TUNN. UNDERGR. SP TECH.* **88**, 169–185 (2019).

[26] Idris, M. A., Saiang, D. & Nordlund, E. Stochastic Assessment of Pillar Stability at Laisvall Mine using Artificial neural network. *TUNN. UNDERGR. SP TECH.* **49**, 307–319 (2015).

[27] Wang, Y. et al. Stress and deformation evolution characteristics of Gob-Side Entry retained by roof cutting and pressure relief. *TUNN. UNDERGR. SP TECH.* **123**, 104419 (2022).

[28] Ma, W. W. Study on surrounding rock control technology of roadway in large mining height hard roof face. *Saf. Coal Mines*. **53**, 94–103 (2022).

[29] Yin, S. F., Zuo, A. J., Ma, L. J. & Ren, Y. X. Shi. Surrounding rock stability during gob–side entry driving with narrow coal pillar in medium-thick coal seam. *Coal Eng.* **54**, 90–96 (2022).

[30] Liu, P. Z. et al. Research on reasonable coal pillar width of coal–rock gob–side entry driving in inclined coal seam. *Coal Eng.* **55**, 12–18 (2023).

[31] Wang, P. F. et al. Re-discussion on reasonable position and support technology of entry driven under the gob edge of previous split-level long wall panel. *J. China Coal Soc.* **48**, 593–608 (2023).

[32] Dong, S. Y., Fu, S. X. & Li, R. Optimization of section coal Pillar size and Engineering Practice in large mining Hight Working face with Soft-Thick Mudstone Roof. *Coal Technol.* **42**, 76–80 (2023).

[33] Yang, S., Chen, M., Jing, H., Chen, K. & Meng, B. A. Case Study on large deformation failure mechanism of Deep Soft Rock Roadway in Xin’an Coal Mine, China. *ENG. GEOL.* **217**, 89–101 (2017).

[34] Yang, H. et al. Adaptation Assessment of Gob-Side Entry retaining based on geological factors. *ENG. GEOL.* **209**, 143–151 (2016).

[35] Yang, J. P. Rational width of narrow coal pillar and control technology of surrounding strata at roadway driven along goaf. *J. Liaoning Tech. Univ. (Natural Science)*. **32**, 39–43 (2013).

[36] Zhang, K. X. et al. Determination of the narrow pillar width of gob-side entry driving. *J. Min. Saf. Eng.* **32**, 446–452 (2015).

[37] Xie, G. X., Yang, K. & Chang, J. C. Influenced of coal pillar width on deformation and fracture of gateway surrounding rocks in fully mechanized top-coal caving mining. *J. Liaoning Tech. Univ.* **26**, 173–176 (2007).

[38] Feng, J. C., Ma, N. J., Zhao, Z. Q. & Zhang, H. Yu. Width of narrow coal pillar of roadway driving along goaf at large height mining face in deep mine. *J. Min. Saf. Eng.* **31**, 580–586 (2014).

[39] Zheng, X. G., Yao, Z. G. & Zhang, N. Stress Distribution of Coal Pillar with Gob-Side Entry driving in the process of Excavation & Mining. *J. Min. Saf. Eng.* **29**, 459–465 (2012).

[40] Sun, D. F. & Shang, Q. Study on narrow coal pillar width and surrounding rock control of gob–side entry driving with large mining height and section of 6.8 m in deep coal mine. *Saf. Coal Mines*. **53**, 166–173 (2022).

[41] Sun, F. Y. Instability mechanism and control technology of surrounding rock of gob–side entry with narrow pillar by fully-mechanized caving mining. *Coal Sci. Technol.* **46**, 149–154 (2018).

[42] Zhang, G. C. & He, F. L. Pillar width determination and surrounding rocks control of gob-side entry with large cross-section and fully-mechanized mining. *Rock. Soil. Mech.* **37**, 1721–1728 (2016).

